# Transcriptomic analysis functionally maps the intrinsically disordered domain of EWS/FLI and reveals novel transcriptional dependencies for oncogenesis

**DOI:** 10.18632/genesandcancer.188

**Published:** 2019-02

**Authors:** Emily R. Theisen, Kyle R. Miller, Iftekhar A. Showpnil, Cenny Taslim, Kathleen I. Pishas, Stephen L. Lessnick

**Affiliations:** ^1^ Center for Childhood Cancer and Blood Diseases, The Research Institute at Nationwide Children's Hospital, Columbus, OH, USA; ^2^ Molecular, Cellular, and Developmental Biology Program, The Ohio State University, Columbus, OH, USA; ^3^ Division of Pediatric Hematology/Oncology/Blood & Marrow Transplant, The Ohio State University, Columbus, OH, USA

**Keywords:** Ewing sarcoma, EWS/FLI, RNA-seq, structure-function, intrinsically disordered domains

## Abstract

EWS/FLI is the pathognomic fusion oncoprotein that drives Ewing sarcoma. The amino-terminal EWS portion coordinates transcriptional regulation and the carboxy-terminal FLI portion contains an ETS DNA-binding domain. EWS/FLI acts as an aberrant transcription factor, orchestrating a complex mix of gene activation and repression, from both high affinity ETS motifs and repetitive GGAA-microsatellites. Our overarching hypothesis is that executing multi-faceted transcriptional regulation requires EWS/FLI to use distinct molecular mechanisms at different loci. Many attempts have been made to map distinct functions to specific features of the EWS domain, but described deletion mutants are either fully active or completely “dead” and other approaches have been limited by the repetitive and disordered nature of the EWS domain. Here, we use transcriptomic approaches to show an EWS/FLI mutant, called DAF, previously thought to be nonfunctional, displays context-dependent and partial transcriptional activity but lacks transforming capacity. Using transcriptomic and phenotypic anchorage-independent growth profiles of other EWS/FLI mutants coupled with reported EWS/FLI localization data, we have mapped the critical structure-function requirements of the EWS domain for EWS/FLI-mediated oncogenesis. This approach defined unique classes of EWS/FLI response elements and revealed novel structure-function relationships required for EWS/FLI activation at these response elements.

## INTRODUCTION

Ewing sarcoma is an aggressive pediatric bone cancer characterized by a chromosomal translocation which fuses the 5′ portion of the *EWSR1* gene with the 3′ portion of the *FLI1* gene [1–4]. The resulting pathognomonic fusion protein EWS/FLI functions as an oncogenic transcription factor [1, 5, 6]. The FLI domain contains an ETS family DNA-binding domain (DBD) and the EWS domain harbors well-defined transcriptional activation and repression activity and the ability to recruit co-regulatory partners [6–10]. The EWS portion also confers novel DNA binding properties to FLI, such that the fusion binds repetitive GGAA-microsatellites [11–13]. Ewing sarcoma cells depend upon EWS/FLI expression, lack additional ubiquitous genetic mutations, and show widespread epigenomic and transcriptomic alterations driven by the fusion protein [14–18]. These features make Ewing sarcoma an ideal model to study the interplay between epigenomic and transcriptional regulation underlying oncogenesis, particularly in mutationally quiet pediatric cancers.

Transcriptional regulation by EWS/FLI is multi-faceted, affecting thousands of genes [14, 19]. Both gene activation and repression are critical for transformation and direct targets are regulated from both nearby (“promoter-like”) and distant (“enhancer-like”) EWS/FLI-bound loci [8, 9, 14, 20, 21]. These sites include both high affinity ETS motifs, as well as the GGAA-microsatellite repeats uniquely accessible to EWS/FLI [9, 13, 22, 23]. This requires EWS/FLI to engage different co-regulatory complexes, and we hypothesize the ability of this transcription factor to interact with diverse co-regulatory modules arises intrinsically from distinct features within the EWS domain.

How this is accomplished is poorly understood and addressing this key question has been hampered by the repetitive nature of the EWS domain. The EWS region displays low complexity and intrinsic disorder, containing repetitions of a degenerate hexapeptide motif (DHR) comprised of a consensus sequence of SYGQQS, with tyrosine in position 2 absolutely conserved [24, 25]. There are several models of EWS domain function, all of which hinge on the primacy of 37 tyrosine residues driving molecular assembly. One model proposed the EWS domain acts like “molecular Velcro,” with the aromaticity of the tyrosine residues making intermolecular contacts with important co-regulators [24, 25]. In this model, mutating a small number of tyrosines minimally impacts function, while changing a majority of tyrosine residues dramatically reduces intermolecular interactions.

Other models of EWS domain function focus on the importance of tyrosine residues in driving intramolecular interactions, resulting in local phase separation or EWS polymerization [10, 26–30]. These assemblies further interact with the transcriptional machinery, including the C-terminal domain of RNA polymerase II (RNAPII) [26, 30]. Indeed, recent work shows phase separation enables EWS/FLI to both bind GGAA-repeats and recruit chromatin regulators, like BAF complexes and p300 [10]. These co-regulators locally remodel chromatin to promote *de novo* enhancer formation and gene activation [9, 10, 21, 23]. Indeed, small fragments of the EWS portion limited to prion-like [G/S]Y [G/S]Q “SYGQ” domains, either SYGQ1 (EWS domain residues 36-72) or SYGQ2 (EWS domain residues 201-264), fused to FLI were sufficient for phase separation and corresponding gene activation at a subset of known microsatellite-activated targets [10]. It is currently unknown whether activation from a small number of microsatellites is sufficient for transformation. A minimal transforming transcriptional signature has not been established and whether SYGQ-FLI mutants recapitulate EWS/FLI activity at repressed and non-microsatellite targets remains untested.

**Figure 1:**
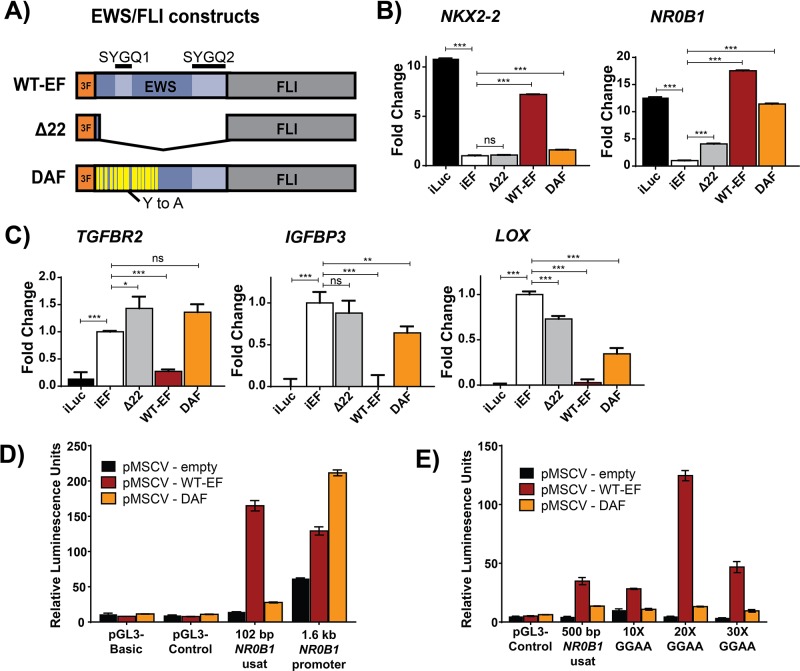
DAF regulation at EWS/FLI target genes is context-dependent **A.** Schematic depicting EWS/FLI cDNA constructs introduced to cells. 3F denotes the N-terminal 3XFLAG tag. The EWS domain is blue and the FLI domain is gray. SYGQ subdomains are depicted in lighter shade, and tyrosine (Y) to alanine (A) mutations in DAF are represented in yellow. **B,C**) qRT-PCR data showing the fold change following rescue with EWS/FLI constructs at **B.** EWS/FLI-activated genes *NKX2-2* and *NR0B1* and **C.** EWS/FLI-repressed genes *TGFBR2*, *IGFBP3*, and *LOX*. Data shown depicts 3 technical replicates and is a representative sample of data acquired from 3 biological replicates. Mean and standard deviation are shown P-values were determined using a Tukey's honest significance test for multiple comparisons. *p < 0.05, **p < 0.01, ***p < 0.001, ns = not significant. **D,E**) Luciferase reporter assays show EWS/FLI and DAF activation from **D.** the isolated NR0B1 microsatellite paired with the SV40 or the native promoter and **E.** artificial microsatellites upstream of the SV40 promoter. Data are plotted as the ratio of firefly to *Renilla* luminescence units to control for transfection efficiency, and show 3 technical replicates of a representative result from 3 independent experiments.

Prior attempts to map distinct functional domains of EWS/FLI failed to identify constructs with partial function. Assayed deletion mutants either 1) retained complete transcriptional and transforming function or 2) lacked detectable activity [14, 19, 31]. In this study we turned to a mutant of EWS/FLI called DAF, which contains Y to A mutations in the first 17 DHRs of the EWS domain (Figure 1A, [24]). While these mutations resulted in a transcriptionally “dead” EWS domain (when fused to ATF), the DAF mutant contains an intact SYGQ2 domain that we hypothesized would confer activity at GGAA-repeats. Previous characterization of DAF did not assay transcriptional function at microsatellites or evaluate oncogenic capacity in a relevant human cell line; mouse (NIH3T3) genomes contain GGAA-repeats at different loci than in the human genome [24, 32]. We used a combination of knockdown-rescue experiments and GGAA-microsatellite reporter assays to evaluate DAF regulatory activity at GGAA-repeats and other EWS/FLI targets. DAF showed unexpected context-dependent activity in isolated reporter assays so we used transcriptomic analysis integrated with phenotypic data from Ewing sarcoma cells to study this important partially-active EWS/FLI construct. This allowed us to define critical EWS/FLI transcriptional response elements and map response of specific elements to distinct features within the EWS domain.

## RESULTS

### DAF displays context-dependent regulation of the EWS/FLI target gene, *NR0B1*

We first assessed DAF activity against five critical EWS/FLI targets using our established knockdown/rescue (KD/Rescue) system [8, 14]. Briefly, shRNA-mediated depletion of endogenous EWS/FLI (endo-EF) was complemented with cDNA encoding an exogenous wildtype EWS/FLI (WT-EF), a negative control (Δ22) lacking the EWS domain, or mutant of interest (Figure 1A, Supplementary Figure 1A-1B). We use the A673 Ewing sarcoma cell line for these experiments, as it is the only cell line to reliably proliferate following depletion of EWS/FLI and subsequent rescue, in our experience. Expression of microsatellite-activated target genes (*NKX2-2* and *NR0B1*), and other directly-repressed targets (*TGFBR2*, *IGFBP3*, and *LOX*) were assayed following rescue (Figure 1B-1C). Surprisingly, DAF failed to recapitulate WT-EF activity at *NKX2-2* and *TGFBR2*, but partially rescued activation of *NR0B1* and repression of *LOX* and *IGFBP3*. Both *NKX2-2* and *NR0B1* are activated by EWS/FLI-bound microsatellites, though the *NR0B1* microsatellite is a “promoter-like” microsatellite, while *NKX2-2* possesses a distal “enhancer-like” microsatellite.

To better understand the different response of different GGAA-repeat-regulated genes to DAF, we utilized a luciferase reporter with isolated GGAA-repeat elements upstream of luciferase in HEK293-EBNA cells transfected with WT-EF or DAF (Supplementary Figure 1C). We first compared DAF activity at the *NR0B1* microsatellite isolated upstream of a minimal SV40 promoter to the same microsatellite within 1.6 kb of the endogenous *NR0B1* promoter (Figure 1D). DAF showed minimal activation from the isolated microsatellite, but activity comparable to, or even greater than, WT-EF when the whole promoter was used. The endogenous *NR0B1* microsatellite has a GGAA-repeat length that falls into an empirically-defined “sweet spot” for EWS/FLI [20, 33, 34]. We wondered whether the mutations in DAF altered its preferred “sweet spot” and tested a small panel of artificial microsatellites with 10X, 20X, and 30X GGAA-repeats. WT-EF showed activity across all microsatellite motifs, with peak activity at the 20X GGAA sweet spot, as expected. Surprisingly, DAF showed consistently weak activation at microsatellites with ≥ 20 repeats (Figure 1E). These data suggest the full *NR0B1* promoter contains additional elements which influence gene responsiveness to EWS/FLI and that DAF activity is greatly impaired at microsatellites lacking these elements.

### Transcriptional profiling reveals EWS/FLI depletion is only partially rescuable

**Figure 2:**
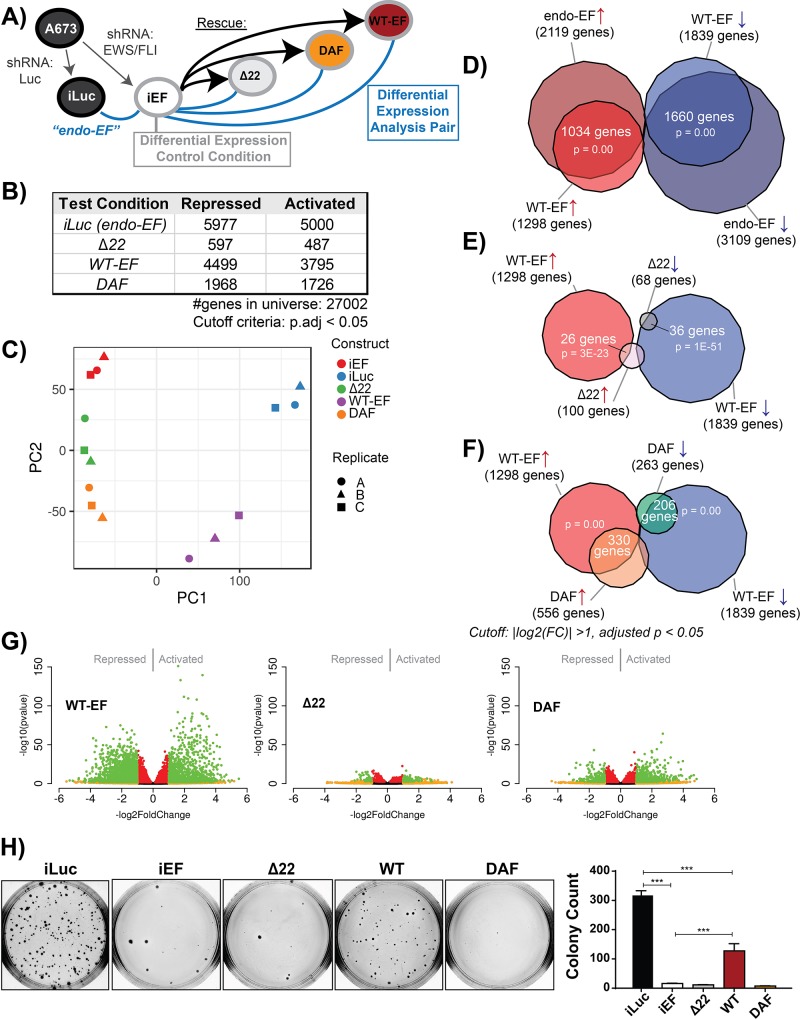
Partial rescue of EWS/FLI global transcription by DAF is insufficient for transformation **A.** Experimental schematic depicting study design for a single replicate. Briefly, cells were transduced with shRNA targeting either luciferase as a control (iLuc) or EWS/FLI (iEF). iEF cells were subsequently rescued with different constructs. After 10 days of selection, these cells were harvested for protein or RNA or seeded into agar. For data analysis purposes, iEF is used as the control to build a transcriptional profile for each EWS/FLI construct. **B.** Table showing number of genes differentially expressed for each construct as compared to iEF, using a adjusted *p* < 0.05 cutoff (Benjamini-Hochberg correction). **C.** Principle component analysis (PCA) plot of the transcriptional profiles of different test conditions. Principle component 2 on the y-axis is plotted against principle component 1 on the x-axis. Different cell conditions are depicted by color and different replicates are represented with different shapes. **D-F)** Venn diagrams comparing the overlapping genes differentially expressed by **D.** WT-EF and endogenous EWS/FLI, **E.** WT-EF and Δ22, and **F.** WT-EF and DAF. Genes included in these analyses met a cutoff of |fold change| > 2 and adjusted *p* < 0.05 (Benjamini-Hochberg). P-values were determined using a Chi-square test. **G.** Volcano plots of differentially expressed genes comparing rescued cells against unrescued cells. The -log(p-value) is plotted against the -log2(FoldChange) for each gene. Genes meeting a cutoff of |log2(FoldChange)| > 1 are shown in yellow. Genes meeting a cutoff of adjusted *p* < 0.05 are shown in red. Genes meeting both cutoffs are shown in green. **H.** Colony formation assays of cells used for transcriptional profiling. Representative agars are shown on the left with corresponding colony counts on the right. Counts are depicted as mean and standard deviation of 3 technical replicates, and these are a representative sample of 3 independent experiments. P-values were determined using a Tukey's honest significance test for multiple comparisons. *p < 0.05, **p < 0.01, ***p < 0.001, ns = not significant.

Our quantitative RT-PCR (qRT-PCR) and reporter data showed that DAF retained activity in a context-specific manner. To evaluate this effect more broadly, we evaluated genome-wide transcriptional function using RNA-seq. The experimental schema is depicted in Figure 2A. Briefly, we used KD/rescue to generate A673 cells under transcriptional control of various EWS/FLI constructs. To create transcriptional profiles for each construct, we used the EWS/FLI-depleted condition, iEF, as a control and compared gene expression patterns driven by each construct against this signature. This approach resulted in transcriptional profiles for WT-EF, Δ22, and DAF. We also included cells which have no depletion of EWS/FLI, iLuc, to internally define the baseline transcriptional profile for endo-EF. This analytical strategy allowed us to characterize various unreported features of the KD/rescue system and transcriptomic-level responsiveness to structural alterations in the EWS domain.

While anchorage-independent growth and qRT-PCR assays from this KD/rescue system have been previously reported [8, 14], and the transcriptional changes which occur with EWS/FLI depletion are well-characterized [9, 14, 19, 35, 36], little has been done to characterize the global transcriptional features rescued with WT-EF. Analysis of these experiments required batch-normalization with ComBat [37], so we first asked whether this new analytical step affected the EWS/FLI transcriptional signature. We compared our batch-normalized endo-EF transcriptional signature with that generated from previously published raw data for EWS/FLI-depleted A673 cells [19] using the same differential expression analytical pipeline, but without batch normalization. Using an adjusted *p*-value < 0.05 as a cutoff we detected 1324 differentially activated genes in the previously published data and 2119 activated genes from the current dataset, with 961 genes overlapping (*p* < 0.0001, Supplementary Figure 2A). As seen previously, endo-EF drove more gene repression. Prior and current experiments identified 2680 and 3109 repressed genes, respectively, with 1937 common genes (*p* < 0.0001, Supplementary Figure 2A). Plotting the fold change of each gene in the current experiment against the fold change in the 2013 dataset shows significant correlation (*R* = 0.93, *p* < 2.2 E-16) between experiments (Supplementary Figure 2B). Confident in the integrity of our baseline endo-EF transcriptional signature, we next compared the genes regulated by endo-EF and those regulated by WT-EF following KD/rescue.

Quantitative RT-PCR showed robust rescue of EWS/FLI target gene regulation by WT-EF at individual genes, like *NR0B1* or *TGFBR2* (Figure 1B-1C). However, the number of colonies formed in soft agar from WT-EF-rescued cells was consistently lower than that from cells without EWS/FLI depletion (See Figure 2H for an example, [14]). This suggests oncogenic capacity is incompletely rescued and the discrepancy between degree of rescue at individual genes and colony formation is consistent, but unexplained. In line with this observation we found that WT-EF rescues most (~75%, Figure 2B), but not all, of the endo-EF transcriptional signature. WT-EF-rescued cells cluster separately from iEF and iLuc, intermediate between the two, in principle component analysis (PCA) (Figure 2C), consistent with incomplete rescue. This reduction in “strength” of the rescue is also visualized in volcano and MA plots, both of which show restricted measures of differential expression in the WT-EF data compared to endo-EF (Supplementary Figure 2C-2D). This decreased magnitude of differential expression in WT-EF-rescued cells is also apparent in Venn analysis. Using a 2-fold change in expression and an adjusted *p*-value < 0.05 as cutoffs, WT-EF rescues ~50% of both EWS/FLI activated and repressed genes, though this overlap is highly significant (*p* < 0.0001, Figure 2D).

### DAF is partially active, but fails to transform

We next compared the transcriptional activity of other EWS/FLI mutants to “full” rescue with WT-EF. Δ22 clusters closely to iEF cells in PCA (Figure 2C). Both the volcano and MA plot for Δ22 show very little differential expression compared to WT-EF, and when using a stricter 2-fold change cutoff for Venn analysis Δ22 only rescues ~2% of genes rescued by WT-EF (Figure 2E, 2G, Supplementary Figure 2D). Interestingly, DAF shows an intermediate phenotype, clustering between Δ22 and WT-EF in PCA and possessing larger measures of differential expression than Δ22 (Figure 2C, 2F, 2G, Supplementary Figure 2D). Activation and repression are both partially rescued with activation more robustly rescued than repression, consistent with our findings in Figure 1. Venn analysis confirmed this, with ~25% of WT-EF activated genes rescued by DAF, while only ~11% of repressed genes are rescued (Figure 2F).

While previous reports showed DAF lacked the capacity to transform NIH3T3 cells [24], we tested the ability of DAF to transform EWS/FLI-depleted Ewing sarcoma cells as a more disease relevant model (Figure 2H). As in prior transformation assays, WT-EF rescued transformation, albeit incompletely, and DAF-rescued cells failed to establish colonies. These data demonstrate the necessity of the N-terminal DHR tyrosines in EWS/FLI-mediated oncogenic phenotypes, like anchorage-independent growth.

### DAF fails to activate transcriptional regulatory genes important for EWS/FLI-mediated oncogenesis

**Figure 3:**
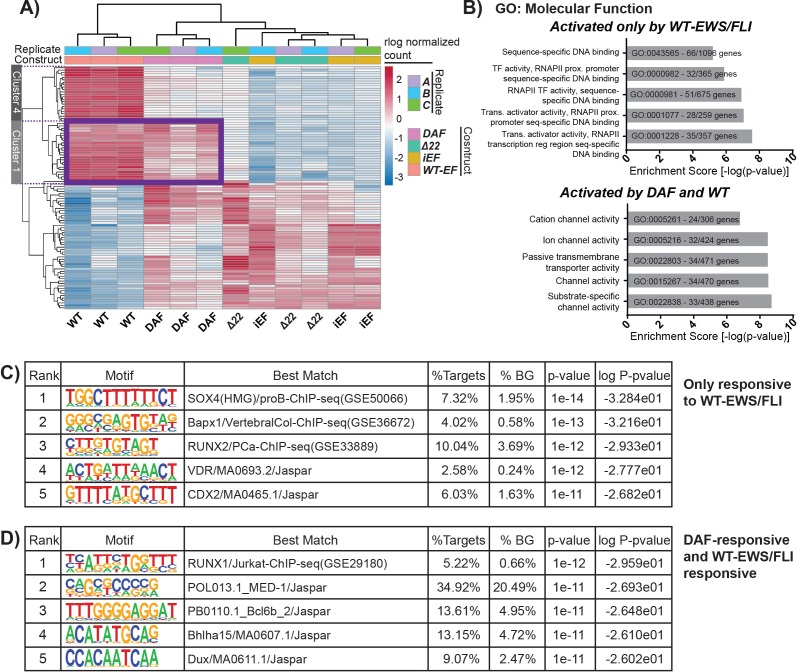
DAF-rescued genes have a distinct function from those rescued only by WT-EF **A.** Heatmap showing unsupervised hierarchical clustering of the 100 most variable genes across samples from RNA-seq analysis. Red depicts activation and blue depicts repression. This analysis identified a cluster of genes regulated by both DAF and WT-EF. **B.** The top five categories from ToppGene functional enrichment analysis are shown for the Gene Ontology: Molecular Function category. Functional enrichment was performed for genes activated either only by WT-EF or by WT-EF and DAF. Genes were included in analysis if they met a cutoff of 2 fold-change and Benjamini-Hochberg adjusted *p*-value < 0.05. **C,D**) Top five enriched motifs for genes activated either only by **C.** WT-EF or by **D.** WT-EF and DAF using HOMER *de novo* motif enrichment analysis of target gene promoters. The letters are color coded by base: green for adenine, red for thymine, blue for cytosine, and yellow for guanine. The size of the letter reflects the prevalence of that base in that particular position in the detected DNA motif. The larger the letter, the more conserved that particular base is in that position across instances of the motif, Genes were included in analysis if they met a cutoff of 2 fold-change and Benjamini-Hochberg adjusted *p*-value < 0.05.

To further understand the difference between genes rescued only by WT-EF and those rescued by both WT-EF and DAF, we performed unsupervised clustering of highly variable genes. This analysis identified a subset of genes commonly activated by DAF and WT-EF, and not responsive to Δ22 (Figure 3A, Cluster 1). We asked whether genes activated only by WT-EF were functionally different from those rescued by DAF. Using ToppGene functional enrichment analysis [38–40] WT-EF-specific activated genes showed strong enrichment for molecular functions associated with the activity of transcription factors, RNAPII regulation, sequence specific DNA binding, and synaptic function (Figure 3B, Supplementary Figure 3A-3C). These genes included known targets of EWS/FLI important for downstream oncogenic activity such as *NKX2-2* [14], *HOXD* genes [41, 42], *GLI1* [43], and *SOX2* [21, 44].

In contrast, DAF-activated genes were associated with ion transport, transmembrane channels, locomotion, and the extracellular matrix (Figure 3B, Supplementary Figure 3A-3C). We further analyzed promoters of the two groups of activated target genes to determine whether particular regulatory motifs were enriched in ways which correlated with gene responsiveness to WT-EF and DAF. HOMER motif analysis of the promoters of rescued genes returned two distinct sets of *de novo* predicted regulatory motifs (Figure 3C-3D, Supplementary Figure 4A-4B). While no one factor stood out as predominantly responsible for either group of genes, some factors previously reported to interact with EWS/FLI, directly or indirectly, stood out, such as RUNX family members [23, 45], E2F3 [46], and PAX7 [47] (Figure 3C-3D, Supplementary Figure 4A-4B). While DAF shows modest repressive function, an analogous block of EWS/FLI-repressed genes rescued by WT-EF and DAF was not readily apparent in the clustering of the most highly variable genes. This may result either from impaired repressive activity at direct targets or indirectly from a lack of activation at transcriptional repressors, like *NKX2-2* and *GLI1*.

### DAF partially rescues GGAA-microsatellite-activated direct targets

To better understand the impact of N-terminal DHR tyrosine to alanine mutations at different types of direct target genes, we paired these transcriptomic data with previously published EWS/FLI localization data [20]. Based on the intact SYGQ2 domain in DAF and the noted activity at *NR0B1*, we first analyzed GGAA-microsatellite-regulated genes. Recent computational analysis by Johnson, *et al.*, identified distinct classes of GGAA-microsatellite regulated genes based on the genomic distance between microsatellite and gene [20]. Differences in the relationship between EWS/FLI binding and subsequent gene regulation were observed between genes with promoter-like proximal (< 5 kb from transcription start) microsatellites and distant enhancer-like (> 5 kb from transcription start) microsatellites [20]. Moreover, promoter- and enhancer-like repressed genes were identified, also with subtle differences between EWS/FLI binding and subsequent regulation [20]. Preliminary analyses of the RNA-seq data showed proximal microsatellite-activated genes *GSTM4* and *FCGRT* were rescued by DAF, in addition to *NR0B1*. We interpreted these data to suggest DAF is therefore able to bind and act at GGAA-microsatellites. However, DAF showed limited activity from isolated microsatellites and failed to rescue *NKX2-2*. *NKX2-2* is activated by a distant, “enhancer-like” microsatellite [44]. We therefore hypothesized that DAF bound GGAA-repeat regions and activated nearby genes, but lacked the critical ability to interact with the chromatin machinery required for distal regulation.

**Figure 4:**
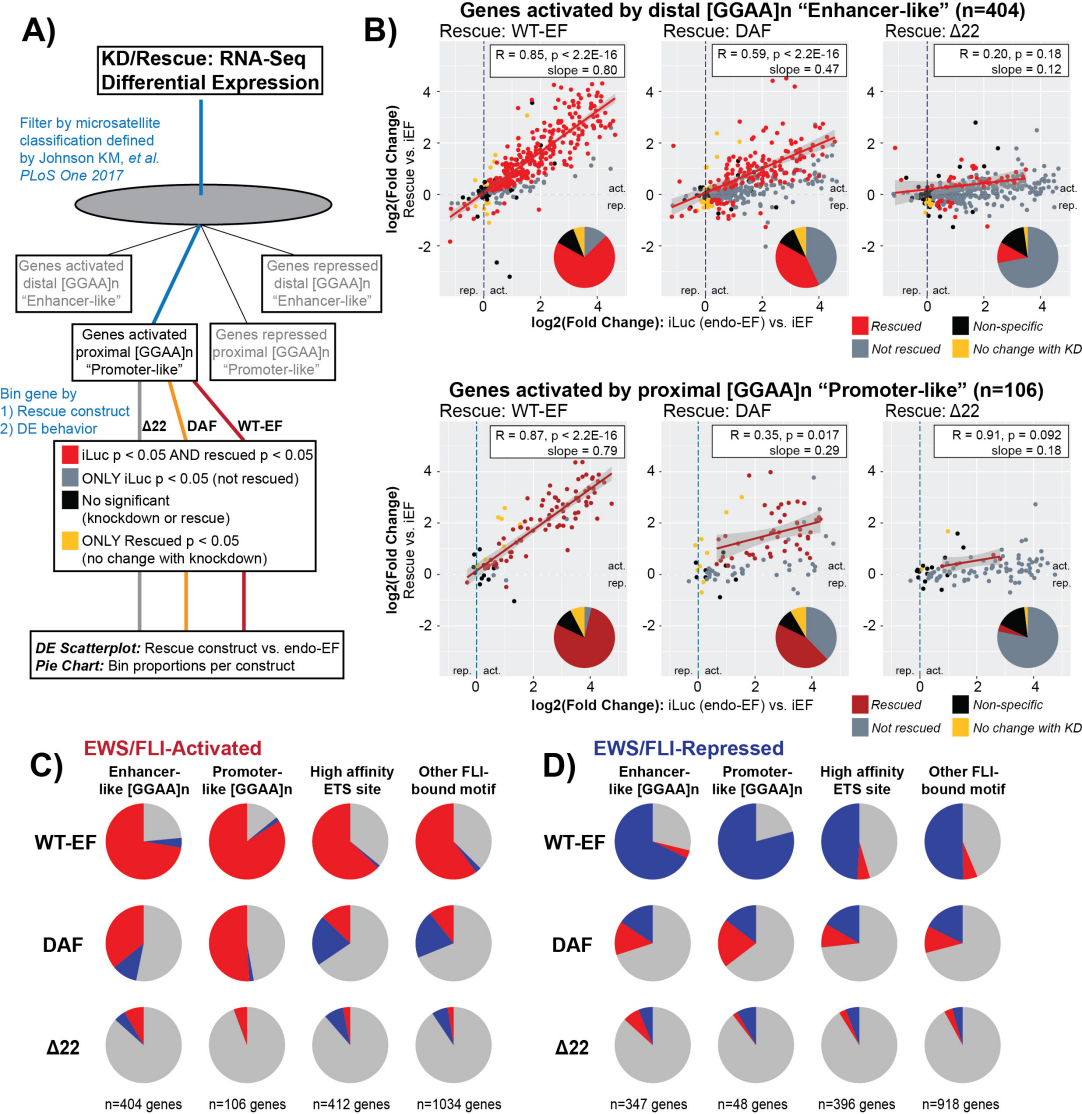
DAF most robustly rescues EWS/FLI function at activated microsatellites **A.** Schematic depicting workflow used to analyze gene rescue at direct microsatellite targets. **B.** Scatterplots depicting construct-specific rescue of activated microsatellites. Data is plotted as log2(FoldChange) of each rescue construct on the y-axis against the log2(FoldChange) of regulation by endogenous EWS/FLI on the x-axis. Dotted lines depict x = 0 and y = 0. Data points in red represent genes whose change in expression was significant in both KD and rescue conditions. Data points in gray indicate genes whose change in expression was significant in only the KD condition. Data points in yellow indicate genes whose change in expression was significant in only the rescue condition. Data points in black indicate genes with no detectable change in expression in these experiments. Significance was defined as Benjamini-Hochberg adjusted *p* < 0.05 with no fold-change cutoff. Lines of best fit were derived from the linear model only for genes with significant changes in both KD and rescue conditions to represent the “volume” of rescued activity. Pearson correlation coefficients and their p-values are depicted with the slope from the linear model in the boxed inset. The pie chart inset for each panel shows the proportion of genes belonging to each functional group for that construct. **C,D**) Compilation depicting the rescue of direct EWS/FLI targets by different constructs at **C.** activated and **D.** repressed target genes. Genes included in this analysis were only those genes with detectable differential expression by endogenous EWS/FLI, and thus represent the “red” and “gray” scatterplot data for activated and “blue” and “gray” scatterplot data for repressed genes. In each pie chart, gray depicts the proportion of genes which are not rescued. Red depicts the proportion of genes that are differentially activated and blue depicts the proportion of genes that are differentially repressed by a given construct.

To address this hypothesis, we examined the construct-specific gene expression profiles for EWS/FLI-bound microsatellite genes in the four categories outlined by Johnson, *et al.*,: 1) enhancer-like microsatellite-activated, 2) promoter-like microsatellite-activated, 3) enhancer-like microsatellite-repressed, and 4) promoter-like microsatellite-repressed (Figure 4A [20]). In Figure 4B, the resulting plots are shown for genes activated by microsatellites, both enhancer-like and promoter-like. We first evaluated whether a given gene, identified as microsatellite-regulated by Johnson, *et al.*, was differentially regulated by endogenous EWS/FLI. If that gene was also rescued by the test construct it is colored in red, with gray signifying failed rescue. Those genes with no significant regulation by endogenous EWS/FLI are colored in black to indicate no change in both knockdown and rescue conditions, or yellow to indicate significant change in expression only with introduction of a rescue construct. Pie chart insets show the proportion of genes comprising each group for each construct. Pearson correlation coefficients and linear models were calculated for rescued genes to assess the quality of the rescue as compared to the degree of activation by endo-EF.

Expression of WT-EF in EWS/FLI-depleted cells was able to rescue ~75% of both distal and proximal microsatellite-activated genes, with significant correlation between activation by endo-EF and WT-EF (*r* = 0.85, slope = 0.80 for distal; *r* = 0.87, slope = 0.79 for proximal) (Figure 4B). In contrast to our prediction that DAF activated proximal, but not distal, microsatellites, we found that DAF was able to activate ~40% of both distal and proximal microsatellite-activated targets. While DAF was able to strongly rescue some genes, the correlation between those genes rescued by DAF and activation by endogenous EF is generally weaker than that seen for rescue with WT-EF (*r* = 0.59, slope = 0.47 for distal; *r* = 0.35, slope = 0.29 for proximal) (Figure 4B).

We next asked whether certain characteristics might make a microsatellite more amenable to rescue by DAF. However, the distributions of 1) maximum number of consecutive GGAA-repeats, 2) total number of GGAA-repeats, and 3) FLI binding enrichment over background for DAF-responsive microsatellites was no different from those distributions observed for WT-EF (Supplementary Figure 5). Δ22 showed minimal rescue at both distal and proximal microsatellite-activated genes (*r* = 0.20, slope = 0.12 for distal; *r* = 0.91, slope = 0.18 for proximal) (Figure 4B).

Instead of supporting a proximal-distal mechanistic dichotomy, these data imply other factors determine whether or not DAF is capable of activating gene expression from a given microsatellite. These data are also consistent with the “molecular Velcro” hypothesis of EWS function, with a partial tyrosine mutant displaying partial function, as measured both by number of genes rescued and the degree of that rescue.

For genes repressed by EWS/FLI-bound microsatellites, WT-EF similarly showed ~75% rescue and good correlation with endo-EF function (*r* = 0.83 and *r* = 0.80 for distal and proximal, respectively) with a slightly decreased degree of rescue (slope = 0.62 and 0.52 for distal and proximal, respectively), suggesting gene repression is a phenotype less amenable to rescue (Supplementary Figure 6). DAF alters expression at ~30% of microsatellite-repressed genes. Surprisingly, many of those genes are paradoxically activated (Supplementary Figure 6). As observed for microsatellite-activated genes, Δ22 showed minimal rescue at microsatellite-repressed genes.

### DAF activity at non-microsatellite direct targets is weak and paradoxical

In addition to GGAA-microsatellites, EWS/FLI binds and regulates genes from consensus ETS sites and other loci [9, 23]. We predicted DAF would show partial rescue of gene activation from these sites, similarly to the activity observed at direct microsatellite targets. Construct-specific scatterplots were generated as above for activated and repressed genes with either high-affinity ETS sites (defined as 5′-ACMGGAARY-3′), or alternative FLI-bound motifs, within 5 kb upstream and 1 kb downstream from transcription start. At these non-microsatellite loci WT-EF was only able to rescue ~50% of target genes (Supplementary Figures 7-8). DAF altered expression of only ~15-25% of these genes, again displaying significant paradoxical activity (Supplementary Figures 7-8). Δ22 showed minimal activity.

To better understand the paradoxical activity of DAF, we took only the genes differentially regulated by endo-EF in this experiment (i.e. excluding data depicted in black and yellow) and, for each construct-motif combination plotted the proportion activated by rescue as red, repressed by rescue as blue, and unchanged by rescue as gray (Figure 4C-4D). For activated genes, WT-EF rescues activation of a majority of genes at each type of response element, with only a small number of incongruently repressed genes. Analogous behavior is seen at repressed genes. DAF shows minimal paradoxical activity at promoter-like activated genes. However, for non-microsatellite activated genes, a majority of the differentially expressed genes are paradoxically repressed. A pattern of ~25-35% “rescue”, but with 30-60% of the genes regulated in the wrong direction characterizes the activity of DAF at all the classes of EWS/FLI direct-targets except microsatellite-activated genes. This paradoxical activity is relatively weak and when stricter fold-change cutoffs (> 2 fold-change) are applied for Venn analysis (Supplementary Figure 9A-9C), these genes are largely excluded.

Interestingly, the direct targets rescued only by WT-EF were again strongly enriched for genes associated with transcriptional regulation and RNAPII recruitment, while DAF- and WT-EF-responsive targets were enriched for receptor binding and ion homeostasis, recapitulating the observation at the whole-transcriptome level (Supplementary Figure 10). Also similar to the global analysis, different types of regulatory motifs were enriched in the promoters of genes directly rescued solely by WT-EF compared to those also rescued following DAF expression (Supplementary Figure 11). These data suggest global transcriptional profiles comprise the downstream consequences of EWS/FLI-mediated regulation of direct target genes.

### Transcriptomic profiling places mut9 function intermediate between DAF and WT-EF

Taken together transcriptomic profiling of DAF showed 1) that DAF most recapitulated WT-EF activity at genes directly activated by EWS/FLI-bound microsatellites, 2) that DAF partially rescues activation from *both* distal and proximal microsatellites, 3) DAF shows weak activity from all other EWS/FLI-bound elements, and 4) the global transcriptional profile for a given mutant is a functional extension of genes regulated directly by EWS/FLI. These data are consistent with a model where the intact SYGQ2 domain in DAF promotes molecular assembly at GGAA-microsatellites, this activity is limited by reduced N-terminal tyrosine content (i.e. reduced molecular Velcro), and the loss of a wildtype amino terminus disrupts the ability to interact with co-regulators at important repressed and non-microsatellite loci. As such, any of the following may cripple the oncogenic capacity of DAF: decreased microsatellite activation, impaired activation from non-microsatellite loci, or global loss of repression.

**Figure 5:**
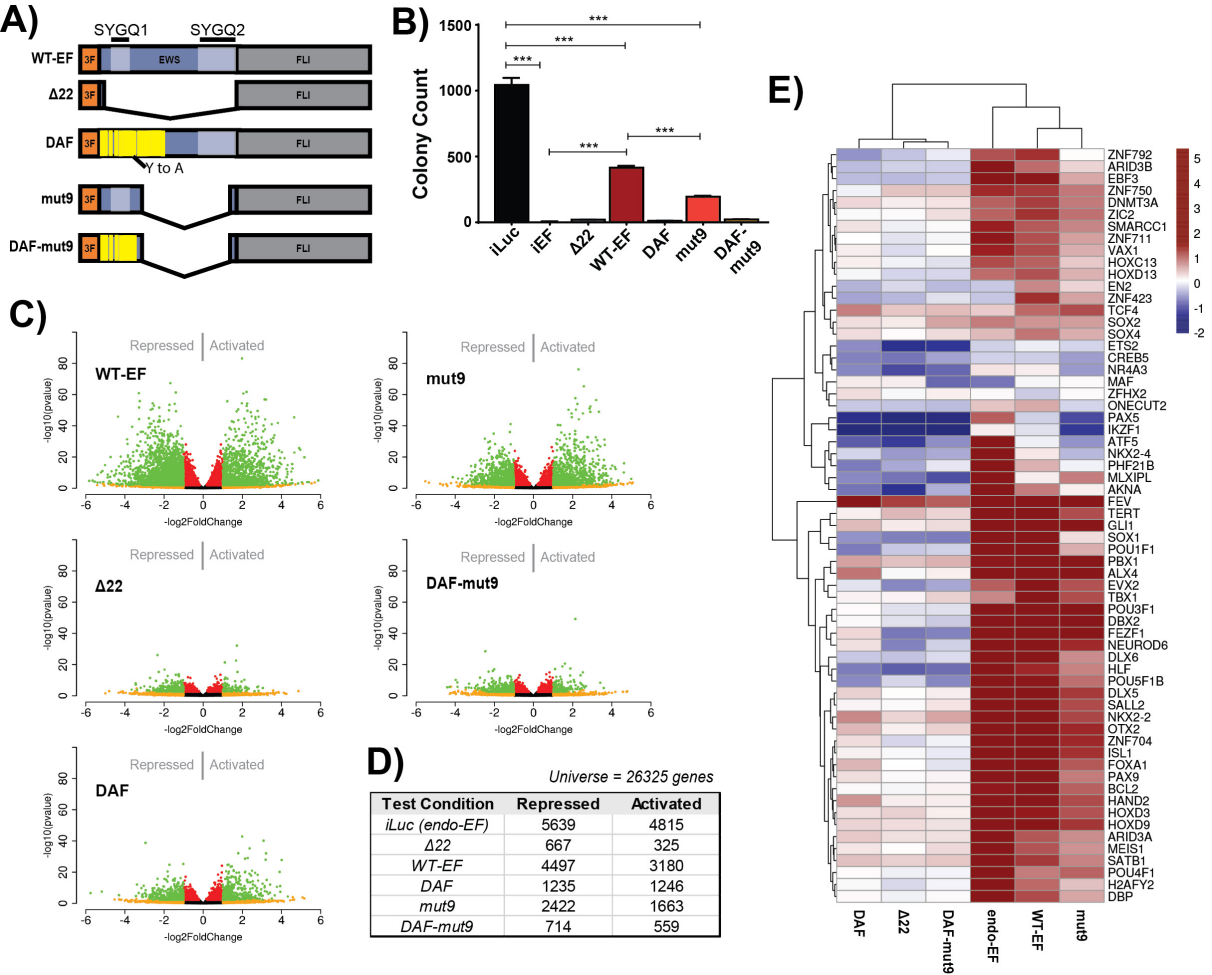
mut9 shows transcriptional activity intermediate to DAF and WT-EF, and transforms cells **A.** Schematic depicting EWS/FLI cDNA constructs introduced to cells. 3F denotes the N-terminal 3XFLAG tag. The EWS domain is blue and the FLI domain is gray. SYGQ subdomains are depicted in lighter blue, and tyrosine to alanine mutations in DAF are represented in yellow. **B.** Colony formation assays of cells used for transcriptional profiling. Counts are depicted as mean and standard deviation of 3 technical replicates, and these are a representative sample of 3 independent experiments. P-values were determined using a Tukey's honest significance test for multiple comparisons. *p < 0.05, **p < 0.01, ***p < 0.001, ns = not significant. **C.** Volcano plots of differentially expressed genes as comparing rescued cells against unrescued cells. The -log(p-value) is plotted against the -log2(FoldChange) for each gene. Genes meeting a cutoff of |log2(FoldChange)| > 1 are shown in yellow. Genes meeting a cutoff of adjusted *p* < 0.05 are shown in red. Genes meeting both cutoffs are shown in green. **D.** Table showing number of genes differentially expressed for each construct as compared to iEF, using a cutoff as adjusted p-value less than 0.05 using a Benjamini-Hochberg correction. **E.** Unsupervised hierarchical clustering of constructs by their respective differential expression of 66 core transcriptional regulator genes identified by GO:0043565 (Figure 3B).

To better understand which transcriptional functions are required for oncongenic phenotypes, we profiled the transcriptome of a “minimal fully functional” EWS/FLI deletion mutant, mut9 [31], and its corresponding DAF mutant (Figure 5A). mut9 contains the first 82 and last 22 amino acid residues of the EWS domain, including an intact SYGQ1 domain, and rescues anchorage-independent growth, critical protein-protein interactions, and recapitulates gene regulation at key target genes [8, 31, 33]. The DAF-mut9 construct contains alanine residues substituted for all of the DHR tyrosines present in mut9, disrupting SYGQ1. We performed three new replicate KD/rescue experiments including both mut9 and DAF-mut9 and first evaluated anchorage-independent growth (Supplementary Figure 12A-12C). WT-EF again reproduced at a reduced number of colonies compared to cells without EWS/FLI depletion. DAF and Δ22 both failed to transform (Figure 5B). Interestingly, though mut9 is considered to contain the minimal EWS required for “full function”, we found decreased colony formation as compared to WT-EF. DAF-mut9 failed to rescue anchorage-independent growth (Figure 5B, Supplementary Figure 12C).

Volcano plots of global DE showed mut9 retained gene activation comparable to WT-EF, but only moderately rescued repression (Figure 5C). DAF-mut9 appeared similar to our negative control, Δ22. Both DAF and Δ22 showed similar profiles as in the prior experiment (Figure 5C). mut9 showed more transcriptional activity (2422 genes repressed, 1663 genes activated) than DAF (1235 genes repressed, 1246 genes activated), but less than WT-EF (4497 genes repressed, 3180 genes activated) (Figure 5D). DAF-mut9 and Δ22 had comparable activity (Figure 5D). Venn analysis confirmed these results, and mut9 clustered between DAF and WT-EF in principle component analysis (Supplementary Figures 12D, 13).

We next compared the function of genes activated only by transforming constructs, WT-EF and mut9, with those also activated by DAF. Gene ontology analysis again showed transcriptional regulatory functions and neuronal differentiation enriched in genes rescued by transforming constructs, while DAF-responsive targets were again enriched for ion channel activity and cell signaling (Supplementary Figures 14, 15). We further performed unsupervised clustering of the expression profile of each mutant at 66 transcriptional regulatory genes identified in our first profiling experiment as uniquely responsive to WT-EF. mut9 activated these genes and clustered with endo-EF and WT-EF, while DAF-mut9 clustered with DAF and Δ22, suggesting activation of these genes correlates with the transforming capacity of an EWS/FLI mutant (Figure 5E).

### Mut9 shows stronger directional fidelity in gene regulation at direct EWS/FLI targets than DAF

**Figure 6:**
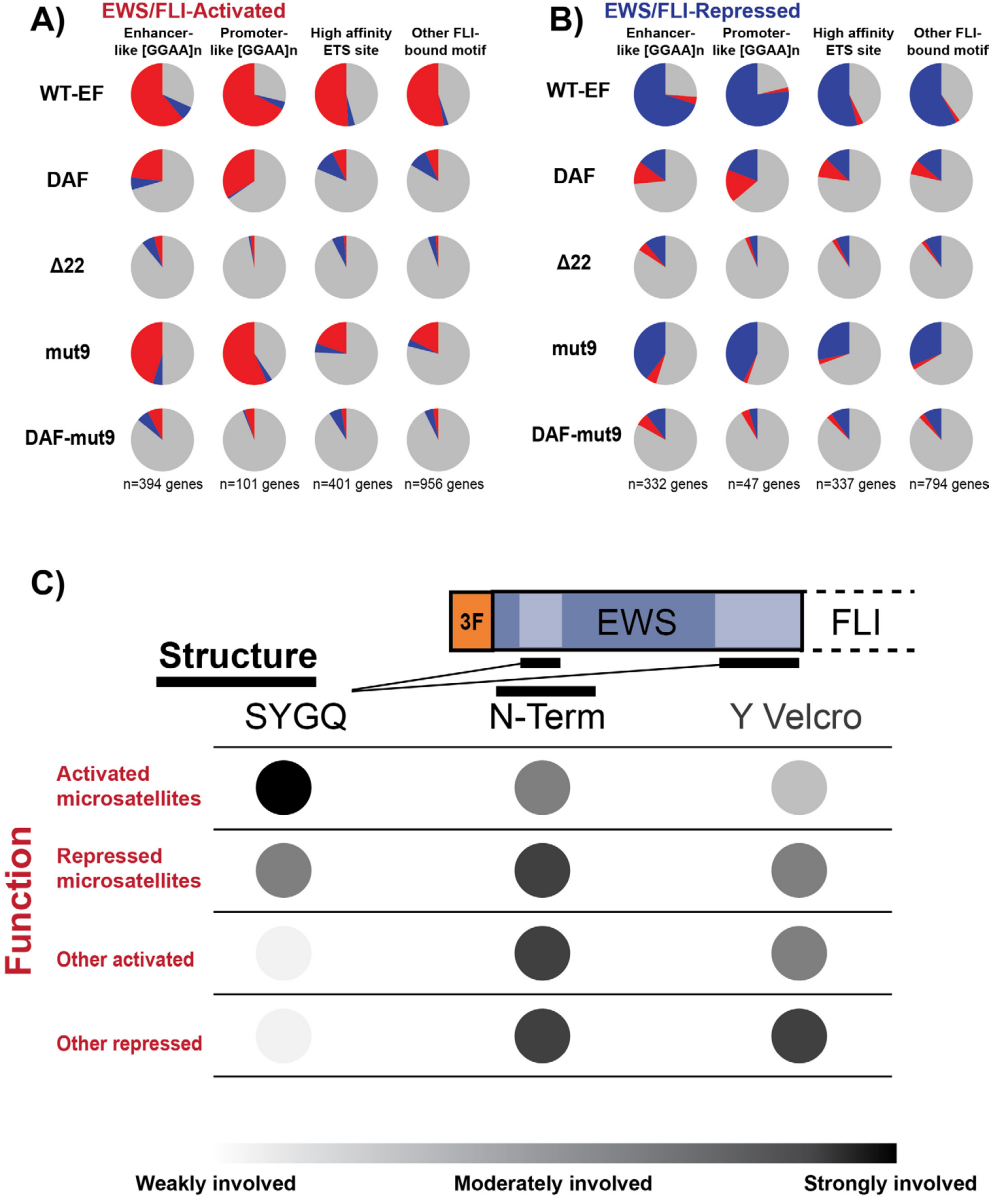
Distinct features of EWS contribute to regulation at different direct EWS/FLI targets **A, B.** Compilation depicting the rescue of direct EWS/FLI targets by different constructs at **A.** activated and **B.** repressed target genes. Genes included in this analysis were only those genes with detectable differential expression by endogenous EWS/FLI, and thus represent the “red” and “gray” scatterplot data for activated and “blue” and “gray” scatterplot data for repressed genes. In each pie chart, gray depicts the proportion of genes which are not rescued. Red depicts the proportion of genes that are differentially activated and blue depicts the proportion of genes that are differentially repressed by a given construct. **C.** Global model of the involvement of different features of EWS in different types of direct EWS/FLI target genes.

As observed across the transcriptome, mut9 activity was intermediate to WT-EF and DAF at direct gene targets, and DAF-mut9 was comparable to Δ22 (Supplementary Figures 16-18). Mut9 most robustly rescued microsatellite target genes, both activated and repressed, with almost complete rescue at promoter-like microsatellite-activated targets (Figure 6A-6B). DAF reproducibly displayed paradoxical activity. We were surprised to see mut9 and DAF regulate similar proportions of genes associated with non-microsatellite response elements (Figure 6A-6B). However, mut9 possessed a higher level of directional fidelity compared to the paradoxical activity of DAF.

These data support the model for EWS domain function in EWS/FLI-mediated gene regulation derived from prior profiling with DAF. The intact SYGQ domain of mut9 contributes to microsatellite activity and an intact amino terminus further reinforces interactions with critical protein complexes, possibly those which determine the direction of gene regulation. However, the reduced overall number of tyrosine residues leads to a reduced ability to interact with co-regulators at non-microsatellite loci, resulting in only ~25% transcriptional activity at these targets. This reduction in direct gene regulation is echoed at the global level, with fewer total differentially expressed genes and a moderately reduced ability to form colonies in agar. A model depicting the dependence of different types of EWS/FLI-mediated transcriptional regulation on EWS domain features is shown in Figure 6C. SYGQ motifs were critically important for activation from GGAA-repeats and moderately important at repressed microsatellites. However, gene regulation from non-microsatellite targets seemed largely agnostic of SYGQ inclusion relying more on the total tyrosine Velcro and intact amino terminus. The amino terminus of the EWS domain was required to maintain the directional fidelity of transcriptional regulation across repressed genes and at non-microsatellite activated targets.

## DISCUSSION

Transcriptomic profiling showed DAF is a partially functional EWS/FLI mutant, representing the first EWS/FLI variant reported to possess partial function. These data were paired with comparable data for wildtype, “minimally functional,” and dead EWS/FLI constructs to map distinct transcriptional regulatory functions to structural features in the EWS domain. Incorporating phenotypic readouts for oncogenic transformation revealed new features of EWS/FLI-mediated transcriptional regulation critical for oncogenesis. This approach accounted for more complexity than accommodated by isolated reporter assays and spot-checking a handful of target genes. Moreover, we revealed an underappreciated context-dependence for EWS/FLI-mediated regulation, the further study of which will be important to better understand EWS/FLI function and design new ways to therapeutically disrupt oncogenesis.

Recent computational analysis by members of our group detailed the relationship between GGAA-repeat characteristics and subsequent gene regulation [20]. They concluded different biochemical mechanisms determine EWS/FLI-responsiveness of a given microsatellite depending on the distance to the target gene [20]. Microsatellites are generally considered important for gene activation, but EWS/FLI bound several GGAA-repetitive regions associated with gene repression, highlighting the mechanistic diversity of microsatellites as EWS/FLI-response elements [20]. The data presented here both reinforce these findings and suggest additional regulatory subtleties. Promoter-like microsatellite target genes were more amenable to rescue than genes with enhancer-like motifs, at both activated and repressed targets. Contrary to our early prediction that the DAF mutations may impair function specifically at enhancer-like motifs, DAF activated a similar proportion of WT-EF-activated microsatellites, regardless of distance to the target gene, suggesting other local factors determine the functional activity of DAF, as seen in our initial *NR0B1* promoter *vs*. isolated microsatellite reporter assay.

We were surprised to see WT-EF rescued repressed microsatellite targets comparably to activated microsatellite targets. While these response elements have been predicted computationally [20], they have not been studied experimentally. The data presented here support repression from GGAA-repeats as a *bona fide* EWS/FLI function. Some models of EWS/FLI-mediated regulation include passive displacement of endogenous ETS transcription factors [9]. However, ETS proteins lack the ability to bind GGAA-repeats under normal conditions, suggesting an active role for EWS/FLI in repression from these sites [11, 23, 48]. Moreover, the discrepancy between mut9 functioning with high fidelity at repressive microsatellites and DAF functioning paradoxically to activate a significant portion of these genes suggest features of the amino terminus of EWS/FLI are critical for EWS/FLI function here. Indeed, we have previously shown the N-terminal region of EWS is sufficient to recruit the NuRD-LSD1 complex to EWS/FLI-mediated repressed targets, and that this recruitment and repression is lost by the EWS-deficient Δ22 [8]. These data also imply different molecular assemblies (i.e. activating *vs*. repressive) interact with different EWS/FLI-bound microsatellites. How such specificity is accomplished when molecular assembly is driven by local phase separation of a disordered and repetitive domain remains an unanswered question.

Non-microsatellite EWS/FLI targets also showed interesting patterns of rescue in this study. On the whole, these motifs were rescued by WT-EF in lower proportions than GGAA-microsatellite regulated targets. Mut9 activity at these loci was only half that of EWS/FLI, and DAF regulated a similar number of genes as mut9, but often acted in the wrong direction. Though mechanisms related to phase separation at microsatellites have recently been reported, the behavior of other important targets support an EWS-as-molecular-Velcro model. At non-microsatellite loci, EWS/FLI must interact with regulatory proteins already present and recruit other co-regulatory units in a context-dependent manner [23]. The data presented here demonstrate that both the wildtype amino terminus and the total number of tyrosines are important for EWS/FLI function at these loci (Figure 6C).

Interpretation of the transcriptomic data in light of colony forming assays further illuminate how EWS/FLI-mediated transcriptional regulation contributes to oncogenesis. Notably, re-introduction of WT-EF rescued an incomplete majority (~80%) of EWS/FLI target genes. This decrease is reflected in the diminished oncogenic capacity of these cells compared to those lacking EWS/FLI depletion. Though mut9 is considered a “fully functional” construct [8, 31, 33], it rescued an even smaller proportion of target genes, particularly at non-microsatellite targets, and formed fewer colonies. These data highlight the importance of both non-microsatellite gene regulation and total tyrosine content for EWS/FLI oncogenic function. The reasons why rescued transcriptional regulation by WT-EF is incomplete is unknown. One possibility is that depletion of endogenous EWS/FLI permits alterations within the regulatory landscape of the cell that are irreversible following re-introduction of EWS/FLI. This model predicts WT-EF to show progressively decreased capacity for rescue as the time elapsed increases between knockdown and rescue.

We additionally uncovered a set of activated genes involved with sequence-specific transcriptional regulation (i.e. transcription factors) which appear to correlate with oncogenic capacity. Some of these genes are known downstream effectors of EWS/FLI, such as *NKX2-2* [14], *HOX* cluster genes [41, 42], *GLI1* [43], and *SOX2* [21, 44]. Core transcriptional regulatory circuits (CRCs) drive oncogenic transcription in other pediatric cancers, like neuroblastoma [49, 50] and rhabdomyosarcoma [51], but a similar analysis has not been reported for Ewing sarcoma. Indeed, there is evidence that the transcriptional profiles of different Ewing sarcoma cell lines and primary tumors are highly heterogeneous [52]. Regardless of whether CRC transcription factors are involved in Ewing sarcoma, transforming mutants were better able to alter the transcriptional landscape through activation of multiple transcription factors involved in varied developmental pathways.

The data presented herein suggest the differential activity of structural EWS/FLI mutants at EWS/FLI response elements is, in part, determined by the ability of such mutants to interact with other local regulatory factors. It is possible the regulatory regions of EWS/FLI-activated transcription factor genes are flagged in a cell- and context-specific manner which preferentially responds to EWS/FLI, but not DAF. However, little is known about the cell-specific upstream determinants of cell susceptibility to transformation by an oncogenic transcription factor. Further investigation will be required to determine how these context-dependent regulatory landscapes are defined and how they vary, both within a single cell and more broadly in Ewing sarcoma.

One limitation of this study is the use of a single cell line, A673. Similar methods have been reported in the EWS502 cell line, however these reagents did not work reliably in our hands [23]. We have made several attempts to develop a secondary experimental system for knockdown and rescue with limited success. Our experience is that while knockdown can occasionally be achieved prior to rescue, it is remarkably inconsistent and unstable following rescue, particularly in cells “rescued” with empty vector, a critical control condition. The reason A673 cells are uniquely tolerant to loss of EWS/FLI remain poorly understood. These cells possess a V600E mutated BRAF, however, RNAi-mediated depletion of BRAF or treatment with vemurafenib shows limited impact on cell behavior [53–55]. Nonetheless, expanding the repertoire of robust systems to meaningfully interrogate the function of EWS/FLI remains an ongoing challenge for the field.

In conclusion, we observed context-dependent and partial activity of DAF in preliminary reporter assays, spurring the use of transcriptomic profiling to map structure-function dependencies within the EWS domain of EWS/FLI. This approach revealed new complexities in EWS/FLI transcriptomic regulation and we used EWS/FLI localization to identify distinct EWS/FLI response elements implicated in oncogenic transcription. Structural mapping showed the amino terminal portion of EWS to be critically important for gene repression, as well as for gene activation from non-microsatellite loci. Total DHR tyrosine content is also important with evidence for both “molecular Velcro” and phase separation involved in EWS/FLI-mediated transcriptional regulation (Figure 6C). Coupling transcriptomic profiling with readouts of relevant biology, such as anchorage-independent growth, represents a new paradigm to better model the complex transcriptional regulatory requirements that must be met by oncogenic fusion transcription factors.

## MATERIALS AND METHODS

### Constructs and retroviruses

The luciferase-RNAi (iLuc), EWS/FLI-RNAi (iEF-2), 3X-FLAG EWS/FLI, 3X-FLAG Δ22, and 3X-FLAG mut9 are previously described [14, 56, 57]. The DAF and mut9-DAF constructs were subcloned into the Murine Stem Cell Virus (pMSCV) backbone (with hyrgomycin resistance) by inserting gene blocks (IDT gBlocks) containing the appropriate tyrosine mutations between the NotI site after the 3X-FLAG tag and the BamHI site in the EWS domain of the 3X-FLAG EWS/FLI vector. shRNA and cDNA expression constructs were packaged into replication deficient retrovirus as previously described [6]. The pGL3-basic and pGL3-promoter vectors were purchased from Promega. The NR0B1 1.6 kb promoter [11], 102 bp NR0B1 microsatellite [12], the 500 bp NR0B1 microsatellite [11], 10X GGAA, 20X GGAA, and 30X GGAA firefly luciferase reporter vectors are previously described [11, 33]. The renilla luciferase vector used is as previously described [12].

### Antibodies

The following antibodies were used for immunodetection: M2-anti-FLAG (Sigma F3165), anti-FLI1 (Abcam ab15289), anti-Lamin B1 (Abcam ab16048), anti-H3 total (D1H2; Cell Signaling Technology 4499), IRDye® 800CW goat anti-mouse IgG (LI-COR Biosciences 926-32210), IRDye® 800CW goat anti-rabbit IgG (LI-COR Biosciences 926-32211), IRDye® 680LT goat anti-rabbit IgG (LI-COR Biosciences 926-68021), anti-rabbit secondary (HRP Cell Signaling 7074).

### Tissue culture, including knockdown/rescue and selection

HEK293-EBNA cells were maintained in DMEM (Corning Cellgro 10-013-CV) supplemented with 10% fetal bovine serum (Gibco 16000-044), penicillin/streptomycin/glutamine (Gibco 10378-016), and 0.3 mg/mL geneticin (Gibco 10131-027). Ewing sarcoma A673 cells were grown in DMEM containing 10% fetal bovine serum, penicillin/streptomycin/glutamine, and sodium pyruvate (Gibco 11360-070). For knockdown of wildtype EWS/FLI and rescue with differing cDNAs, shRNAs were retrovirally delivered on day 1. Selection in 2 μg/mL puromycin (Sigma P8833) occurred on days 2-5. Cells were infected with retrovirus containing the cDNA in the pMSCV-hygro backbone on day 5. Double selection in puromycin and 400 μg/mL hygromycin B (Thermo Fisher 10687010) began on day 6 and continued until day 16. Cells were subsequently harvested for RNA, protein, and seeded into soft agar assays as previously described [58]. For colony formation assays *p*-values were determined using a Tukey's honest significance test for multiple comparisons and the data is available in Supplementary Tables 2.

### RNA isolation and qRT-PCR

Total mRNA was extracted from frozen cell pellets using the RNeasy kit (Qiagen) with the on-column DNase digestion. qRT-PCR was performed using the iTaq™ Universal SYBR® Green One-Step Kit (BioRad 1725150). Fold change was determined relative to the control sample after normalization to the internal control gene *RPL30*. Primer sequences are provided in Supplementary Table 1. *P*-values were determined using a Tukey's honest significance test for multiple comparisons and the data is available in Supplementary Tables 2.

### RNA-seq and data analysis

Libraries were prepared for sequencing per the manufacturer's (Illumina) instructions and sequenced on an Illumina Hi-Seq 4000 to generate 150-bp paired-end reads. STAR 2.5.0c was used to align reads to the human genome build hg19. *htseq-count* (intersection-strict) was used to generate read counts for each gene [59]. Genes were excluded from analysis if they had < 1 count per sample. ComBat was used to adjust the data from each set of replicates for batch effects [37]. DESeq2 was used for differential expression analysis of ComBat-adjusted counts [60]. Volcano plots, principle component analysis, and heatmaps were generated in R. Heatmaps were generated using *pheatmap* with unsupervised hierarchical clustering using the complete linkage method. Scatterplots were generated using *ggplot2*. Pearson correlation coefficients were determined. Slopes were derived from the linear model (*lm*) function in R. Venn diagrams were generated using VennMaster and *p*-values were calculated using a Chi-square test. HOMER findMotifs.pl [61] was used to find motifs of either 8, 10, or 12 bp enriched in the promoters of defined gene lists. Promoters were defined at 2 kb upstream of transcription start and 500 bp downstream from transcription start. Promoters of genes in the test list were compared against all other promoters as the background. ToppGene was used with the default settings for functional enrichment [38–40].

### Data availability

The data discussed in this publication have been deposited in NCBI's Gene Expression Omnibus and are accessible through GEO Series accession number GSE122537 (https://www.ncbi.nlm.nih.gov/geo/query/acc.cgi?acc=GSE122537). These data have also been deposited in NCBI's Sequencing Read Archive through SRA accession numbers SRP168620 and SRP168621.

### Protein isolation

Nuclear proteins were isolated from fresh cell pellets resuspended in 500 μL hypotonic buffer (20 mM HEPES [pH 8.0], 10% glycerol, 10 mM NaCl, 1.5 mM MgCl2, 1 mM EDTA, and 1 mM DTT) with protease inhibitor for 15 minutes. 12.5 μL IGEPAL^®^ CA-630 (Sigma I8896) was added (final concentration 0.5%) and cells vortexed vigorously for 10 seconds to lyse cytoplasm. Nuclei were pelleted at 1000 rcf for 5 minutes at 4°C and washed in 20 mM HEPES [pH 8.0], 10% glycerol, 140 mM NaCl, 1.5 mM MgCl_2_, 1 mM EDTA, 1 mM DTT, and 1% IGEPAL^®^ CA-630 with protease inhibitor. Nuclei were pelleted at 1000 rcf for 5 minutes at 4°C, snap frozen, and stored at −80°C. Proteins were extracted by incubation with RIPA + protease inhibitor on ice for 60 minutes with occasional vortexing. Lysates were clarified by centrifugation at max speed for 60 minutes at 4°C. Protein concentration was assessed using a BCA assay (Thermo Fisher 23225).

### Western blot

Protein samples were prepared in 1X SDS loading buffer and boiled for 5 minutes prior to gel loading. Western blots were run on 4-15% or 7.5% BioRad precast tris-glycine gels. Running conditions were 15 minutes at 90V and 55 minutes at 120V. Proteins were blotted onto nitrocellulose using the iBlot^TM^2 (Thermo Fisher) and developed using Odyssey and chemiluminescence detection.

### Luciferase assays

HEK293-EBNA cells were seeded at a density of 5 × 10^4^ cells/well in a 24-well dish. Triplicate wells were seeded per condition. The following day cells were transfected with firefly reporter (250 ng), *Renilla* plasmid, and cDNA construct (500 ng) using TransIT^®^-LT1 (Mirus MIR 2306). Briefly, DNA and TransIT^®^-LT1 were co-incubated in pre-warmed Optimem (Gibco 31985-070) for 20 minutes before addition to cultured cells (500 μL/well). Cells were incubated with transfection mix for 4 hours followed by addition of normal EBNA media (500 μL/well). Transfected cells were maintained for 24 hours and assayed using the Dual-Glo Luciferase Assay System (Promega) and measured on a Promega GloMax^®^ 96 Microplate Luminometer. Firefly luciferase activity was normalized to *Renilla* luciferase activity to normalize for transfection efficiency.

## SUPPLEMENTARY TABLES AND FIGURES





## Abbreviations

ETS: e26 transformation specific a family of transcription factors with common structure
DNA: deoxyribonucleic acid
DBD: DNA-binding domain
N-terminal: amino terminal referring to a protein or peptide chain
C-terminal: carboxy terminal referring to a protein or peptide chain
Endo-EF: endogenous EWS/FLI
WT-EF: wildtype Type IV EWS/FLI expressed from exogenous cDNA
Δ22: mutant of EWS/FLI lacking all but 6 amino acid from the EWS domain expressed from exogenous cDNA
DAF: mutant of EWS/FLI containing 17 tyrosine to alanine mutations in the amino terminal region of the EWS domain of EWS/FLI expressed from exogenous cDNA
mut9: mutant of EWS/FLI lacking residues 83-246 from the EWS domain expressed from exogenous cDNA
DHR: degenerate hexapeptide motif region consensus amino acid sequence SYGQQS
BAF: BRG1-associated factor
SYGQ: domain containing repetitive serine (S)-tyrosine (Y)- glycine (G)-glutamine (Q) motifs
KD: knockdown
cDNA: coding DNA
EBNA: Epstein Barr nuclear antigen
RNA-seq: RNA sequencing
RNAi: RNA interference
shRNA: short hairpin RNA
iLuc: A673 cells expressing a short hairpin RNA targeting luciferase an RNAi control
iEF: A673 cells expressing a short hairpin RNA targeting the 3′UTR of EWS/FLI
qRT-PCR: quantitative reverse transcriptase polymerase chain reaction
PCA: principle component analysis
RNAPII: RNA polymerase II
RNA: ribonucleic acid
kb: kilobase
bp: basepair
MSCV: Murine Stem Cell Virus
