# Supplementary material for: Transcriptomic analysis functionally maps the intrinsically disordered domain of EWS/FLI and reveals novel transcriptional dependencies for oncogenesis
Source: Genes Cancer. 2019 Feb;10(1-2):21–38. doi: 10.18632/genesandcancer.188 (PMC6420793; doi:10.18632/genesandcancer.188)
Supplement: Supplementary file 1 [file ganc-10-021-s001.pdf]

Supplementary Tables and Figure Legends

Table S1. Primer Sequences for qRT-PCR

Gene	Forward	Reverse
RPL30	5'GGGGTACAAGCAGACTCTGAAG	5'ATGGACACCAGTTTTAGCCAAC
EWS/FLI	5'CAGTCACTGCACCTCCATCC	5'TTCATGTTATTGCCCCAAGC
NKX2-2	5' CTACGACAGCAGCGACAACC	5' GCCTTGGAGAAAAGCACTCG
NR0B1	5' GGGGACCGTGCTCTTTAACC	5' CTGACTGTGCCGATGATGG
TGFBR2	5' CATCTGTGAGAAGCCACAGG	5' TGCACTCATCAGAGCTACAGG
LOX	5' CTGCTCAGATTTCCCCAAAG	5' TGGCATCAAGCAGGTCATAG
IGFBP3	5'CATCAAGAAAGGGCATGCTAA	5'CTACGGCAGGGACCATATTCT

Figure S1. Validation of knockdown and construct expression in A673 and HEK293-EBNA cells

A) qRT-PCR data showing the fold change of EWS/FLI following knockdown of EWS/FLI and rescue with EWS/FLI constructs. Data shown depicts 3 technical replicates and is a representative sample of data acquired from 3 biological replicates. Mean and standard deviation are shown. P-values were determined using a Tukey's honest significance test for multiple comparisons. *$p<0.05$, **$p<0.01$, ***$p<0.001$, ns = not significant. B) Western blot showing depletion of endogenous EWS/FLI and rescue with tagged constructs in A673 cells used for downstream assays. Nuclear lysates were used. Tyrosine to alanine mutations change the physical properties of the EWS domain, resulting in decreased mobility for DAF as compared to WT-EF C) Western blot of whole cell lysate from HEK293 cells for FLAG tag following transfection of either empty vector, WT-EF, or DAF. Tyrosine to alanine mutations change the physical properties of the EWS domain, resulting in decreased mobility for DAF as compared to WT-EF

Figure S2. Evaluation of EWS/FLI transcriptional profile as determined both by KD and rescue

A) Venn diagrams comparing the overlapping genes differentially expressed by EWS/FLI in either previously published data (1) or as determined by the analyses described here. Genes included in these analyses met a cutoff of |fold change| > 2 and adjusted p < 0.05 (Benjamini-Hochberg). P-values were determined using a Chi-square test. B) Scatterplot depicting differential gene expression (log2(foldChange)) in the current dataset on the y-axis against differential expression in previously published data (1). Genes depicted in red are genes which have a Benjamini-Hochberg adjusted p-value < 0.05 in both datasets (i.e. commonly regulated genes). The Pearson correlation coefficient and p-value, as well as a slope derived from the linear model fit to the data are reported in the inset. C) Volcano plots of EWS/FLI-differentially expressed genes as compared to iEF cells. The –log(p-value) is plotted against the –log2(FoldChange) for each gene. Genes meeting a cutoff of |log2(FoldChange)| > 1 are shown in yellow. Genes meeting a cutoff of adjusted p < 0.05 are shown in red. Genes meeting both cutoffs are shown in green. D) MA plots showing the differential expression detected in all tested conditions. The log2(FoldChange) of a gene is plotted on the y-axis against the normalized number of total counts detected for that gene on the x-axis. Data points are red is they have a Benjamini-Hochberg adjusted p < 0.05.

Figure S3. Functional Enrichment of WT-only activated and DAF-activated genes

A-C) Top five categories from ToppGene functional enrichment analysis from the (A) Gene Ontology: Biological Process, (B) Domain, or (C) Pathway category for genes activated either only by WT-EF or by WT-EF and DAF. Genes were included in analysis if they met a cutoff of 2 fold-change and Benjamini-Hochberg adjusted p-value < 0.05.

Figure S4. Additional enriched motifs detected by HOMER for DAF-activated and WT-only activated genes at the transcriptomic level

A,B) Additional enriched motifs for genes activated either by (A) WT-EF and DAF or (B) only by WT-EF using HOMER de novo motif enrichment analysis of target gene promoters. Genes were included in analysis if they met a cutoff of 2 fold-change and Benjamini-Hochberg adjusted p-value < 0.05.

Figure S5. GGAA microsatellites rescued by DAF are similar to those rescued by WT-EF

The distribution of maximum consecutive GGAA motifs, total number of GGAA motifs, and total FLI enrichment are statistically indistinguishable for those microsatellites which are rescued by DAF and those which are not. P-values were determined using Kolmogorov-Smirnov's test. (WT-EF: n = 141, DAF: n = 79)

Figure S6. Construct-specific rescue at GGAA microsatellite-repressed targets

Scatterplots depicting construct-specific rescue of activated microsatellites. Data is plotted as log2(FoldChange) of each rescue construct on the y-axis against the log2(FoldChange) of regulation by endogenous EWS/FLI on the x-axis. Dotted lines depict x = 0 and y = 0. Data points in blue represent genes whose change in expression was significant in both KD and rescue conditions. Data points in gray indicate genes whose change in expression was significant in only the KD condition. Data points in yellow indicate genes whose change in expression was significant in only the rescue condition. Data points in black indicate genes with no detectable change in expression in these experiments. Significance was defined as Benjamini-Hochberg adjusted $p < 0.05$ with no fold-change cutoff. Lines of best fit were derived from the linear model only for genes with significant changes in both KD and rescue conditions to represent the “volume” of rescued activity. Pearson correlation coefficients and their p-values are depicted with the slope from the linear model in the boxed inset. The pie chart inset for each panel show the proportion of genes belonging to each functional group for that construct.

Figure S7. Construct-specific rescue at high affinity ETS-regulated targets

A) Schematic depicting workflow used to analyze gene rescue at direct high affinity ETS targets. B) Scatterplots depicting construct-specific rescue of genes containing a FLI-bound high affinity ETS site within 5 kb upstream and 1 kb downstream of TSS. Data is plotted as log2(FoldChange) of each rescue construct on the y-axis against the log2(FoldChange) of regulation by endogenous EWS/FLI on the x-axis. Dotted lines depict $x = 0$ and $y = 0$. Data points in red represent genes whose change in expression was significant in both KD and rescue conditions for activated targets. Blue is used for repressed targets. Data points in gray indicate genes whose change in expression was significant in only the KD condition. Data points in yellow indicate genes whose change in expression was significant in only the rescue condition. Data points in black indicate genes with no detectable change in expression in these experiments. Significance was defined as Benjamini-Hochberg adjusted $p < 0.05$ with no fold-change cutoff. Lines of best fit were derived from the linear model only for genes with significant changes in both KD and rescue conditions to represent the "volume" of rescued activity. Pearson correlation coefficients and their p-values are depicted with the slope from the linear model in the boxed inset. The pie chart inset for each panel show the proportion of genes belonging to each functional group for that construct.

Figure S8. Construct-specific rescue at targets regulated from other proximal FLI-bound motifs

A) Schematic depicting workflow used to analyze gene rescue at other proximal FLI-bound targets. B) Scatterplots depicting construct-specific rescue of genes containing a binding site not classified as a microsatellite or high affinity site within 5 kb upstream and 1 kb downstream of TSS. Data is plotted as log2(FoldChange) of each rescue construct on the y-axis against the log2(FoldChange) of regulation by endogenous EWS/FLI on the x-axis. Dotted lines depict $x = 0$ and $y = 0$. Data points in red represent genes whose change in expression was significant in both KD and rescue conditions for activated targets. Blue is used for repressed targets. Data points in gray indicate genes whose change in expression was significant in only the KD condition. Data points in yellow indicate genes whose change in expression was significant in only the rescue condition. Data points in black indicate genes with no detectable change in expression in these experiments. Significance was defined as Benjamini-Hochberg adjusted $p < 0.05$ with no fold-change cutoff. Lines of best fit were derived from the linear model only for genes with significant changes in both KD and rescue conditions to represent the “volume” of rescued activity. Pearson correlation coefficients and their p-values are depicted with the slope from the linear model in the boxed inset. The pie chart inset for each panel show the proportion of genes belonging to each functional group for that construct.

Figure S9. Venn analysis of rescued transcriptional regulation by construct and target classification

A-C) Venn diagrams comparing the overlapping genes differentially expressed by WT-EF vs. endogenous EWS/FLI, WT-EF vs Δ22, and WT-EF vs DAF at (A) microsatellite regulated genes, (B) high affinity site regulated genes, and (C) other direct targets. Genes included in these analyses met a cutoff of |fold change| > 2 and adjusted $p < 0.05$ (Benjamini-Hochberg). P-values were determined using a Chi-square test.

Figure S10. Functional enrichment of WT-only activated vs DAF-activated direct target genes

A-D) Top five categories from ToppGene functional enrichment analysis from the (A) Gene Ontology: Molecular Function, (B) Gene Ontology: Biological Process, (C) Domain, or (D) Pathway category for genes directly activated either only by WT-EF or by WT-EF and DAF. Genes were included in analysis if they met a cutoff of 2 fold-change and Benjamini-Hochberg adjusted p-value < 0.05.

Figure S11. Enriched motifs detected by HOMER for direct DAF- and WT-activated or direct WT-only activated genes

A,B) Top 5 enriched motifs for genes directly activated either by (A) WT-EF and DAF or (B) only by WT-EF using HOMER de novo motif enrichment analysis of target gene promoters. Genes were included in analysis if they met a cutoff of 2 fold-change and Benjamini-Hochberg adjusted p-value < 0.05.

Figure S12. Validation of knockdown and construct expression in A673 and HEK293-EBNA cells

A) qRT-PCR data showing the fold change of EWS/FLI following knockdown of EWS/FLI and rescue with EWS/FLI constructs. Data shown depicts 3 technical replicates and is a representative sample of data acquired from 3 biological replicates. Mean and standard deviation are shown. P-values were determined using a Tukey's honest significance test for multiple comparisons. *p<0.05, **p<0.01, ***p<0.001, ns = not significant. B) Western blot showing depletion of endogenous EWS/FLI and rescue with tagged constructs in A673 cells used for downstream assays. Nuclear lysates were used. DAF Tyrosine to alanine mutations change the physical properties of the EWS domain, resulting in decreased mobility for DAF as compared to WT-EF and DAF-mut9 as compared to mut9. C) Colony formation assays of cells used for transcriptional profiling. Representative agars are shown at the left. D) Principle component analysis (PCA) plot of the transcriptional profiles of different test conditions. Principle component 2 on the y-axis is plotted against principle component 1 on the x-axis. Different cell conditions are depicted by color and different replicates are represented with different shapes.

Figure S13. Venn analysis of total transcriptional rescue by different constructs

A-G) Venn diagrams comparing the overlapping genes differentially expressed by (A) WT-EF vs Δ22, (B) WT-EF vs DAF, (C) WT-EF vs. mut9, (D) WT-EF vs DAF-mut9, (E) mut9 vs. DAF, (F) mut9 vs. DAF-mut9, and (G) DAF vs. DAF-mut9. Genes included in these analyses met a cutoff of |fold change| > 2 and adjusted $p < 0.05$ (Benjamini-Hochberg). P-values were determined using a Chi-square test.

Figure S14. Functional enrichment of WT/mut9-activated vs DAF-activated target genes

A-B) Top five categories from ToppGene functional enrichment analysis from the (A) Gene Ontology: Molecular Function and (B) Gene Ontology: Biological Process category for genes either activated only by transforming constructs WT-EF and mut9 or by WT-EF, mut9, and DAF. Genes were included in analysis if they met a cutoff of 1.5 fold-change and Benjamini-Hochberg adjusted p-value < 0.10.

Figure S15. Enriched motifs detected by HOMER for direct DAF- and WT-activated or direct WT-only activated genes

A,B) Top 10 enriched motifs for genes directly activated either by (A) WT-EF, mut9, and DAF or (B) only by WT-EF and mut9 using HOMER de novo motif enrichment analysis of target gene promoters. Genes were included in analysis if they met a cutoff of 1.5 fold-change and Benjamini-Hochberg adjusted p-value < 0.10.

Figure S16. Construct-specific rescue at microsatellite-regulated targets: mut9 data

A) Schematic depicting workflow used to analyze gene rescue at microsatellite-regulated targets. B) Scatterplots depicting construct-specific rescue of genes containing a FLI-bound high affinity ETS site within 5 kb upstream and 1 kb downstream of TSS. Data is plotted as log2(FoldChange) of each rescue construct on the y-axis against the log2(FoldChange) of regulation by endogenous EWS/FLI on the x-axis. Dotted lines depict $x = 0$ and $y = 0$. Data points in red represent genes whose change in expression was significant in both KD and rescue conditions for activated targets. Blue is used for repressed targets. Data points in gray indicate genes whose change in expression was significant in only the KD condition. Data points in yellow indicate genes whose change in expression was significant in only the rescue condition. Data points in black indicate genes with no detectable change in expression in these experiments. Significance was defined as Benjamini-Hochberg adjusted $p < 0.05$ with no fold-change cutoff. Lines of best fit were derived from the linear model only for genes with significant changes in both KD and rescue conditions to represent the “volume” of rescued activity. Pearson correlation coefficients and their p-values are depicted with the slope from the linear model in the boxed inset. The pie chart inset for each panel show the proportion of genes belonging to each functional group for that construct.

Figure S17. Construct-specific rescue at high affinity ETS-regulated targets: mut9 and DAF-mut9 data included

A) Schematic depicting workflow used to analyze gene rescue at direct high affinity ETS targets. B) Scatterplots depicting construct-specific rescue of genes containing a FLI-bound high affinity ETS site within 5 kb upstream and 1 kb downstream of TSS. Data is plotted as log2(FoldChange) of each rescue construct on the y-axis against the log2(FoldChange) of regulation by endogenous EWS/FLI on the x-axis. Dotted lines depict $x = 0$ and $y = 0$. Data points in red represent genes whose change in expression was significant in both KD and rescue conditions for activated targets. Blue is used for repressed targets. Data points in gray indicate genes whose change in expression was significant in only the KD condition. Data points in yellow indicate genes whose change in expression was significant in only the rescue condition. Data points in black indicate genes with no detectable change in expression in these experiments. Significance was defined as Benjamini-Hochberg adjusted $p < 0.05$ with no fold-change cutoff. Lines of best fit were derived from the linear model only for genes with significant changes in both KD and rescue conditions to represent the “volume” of rescued activity. Pearson correlation coefficients and their p-values are depicted with the slope from the linear model in the boxed inset. The pie chart inset for each panel show the proportion of genes belonging to each functional group for that construct.

Figure S18. Construct-specific rescue at targets regulated from other proximal FLI-bound motifs: mut9 and DAF-mut9 data included

A) Schematic depicting workflow used to analyze gene rescue at other proximal FLI-bound targets. B) Scatterplots depicting construct-specific rescue of genes containing a binding site not classified as a microsatellite or high affinity site within 5 kb upstream and 1 kb downstream of TSS. Data is plotted as log2(FoldChange) of each rescue construct on the y-axis against the log2(FoldChange) of regulation by endogenous EWS/FLI on the x-axis. Dotted lines depict x = 0 and y = 0. Data points in red represent genes whose change in expression was significant in both KD and rescue conditions for activated targets. Blue is used for repressed targets. Data points in gray indicate genes whose change in expression was significant in only the KD condition. Data points in yellow indicate genes whose change in expression was significant in only the rescue condition. Data points in black indicate genes with no detectable change in expression in these experiments. Significance was defined as Benjamini-Hochberg adjusted $p < 0.05$ with no fold-change cutoff. Lines of best fit were derived from the linear model only for genes with significant changes in both KD and rescue conditions to represent the “volume” of rescued activity. Pearson correlation coefficients and their p-values are depicted with the slope from the linear model in the boxed inset. The pie chart inset for each panel show the proportion of genes belonging to each functional group for that construct.

Supplementary Figure 1.

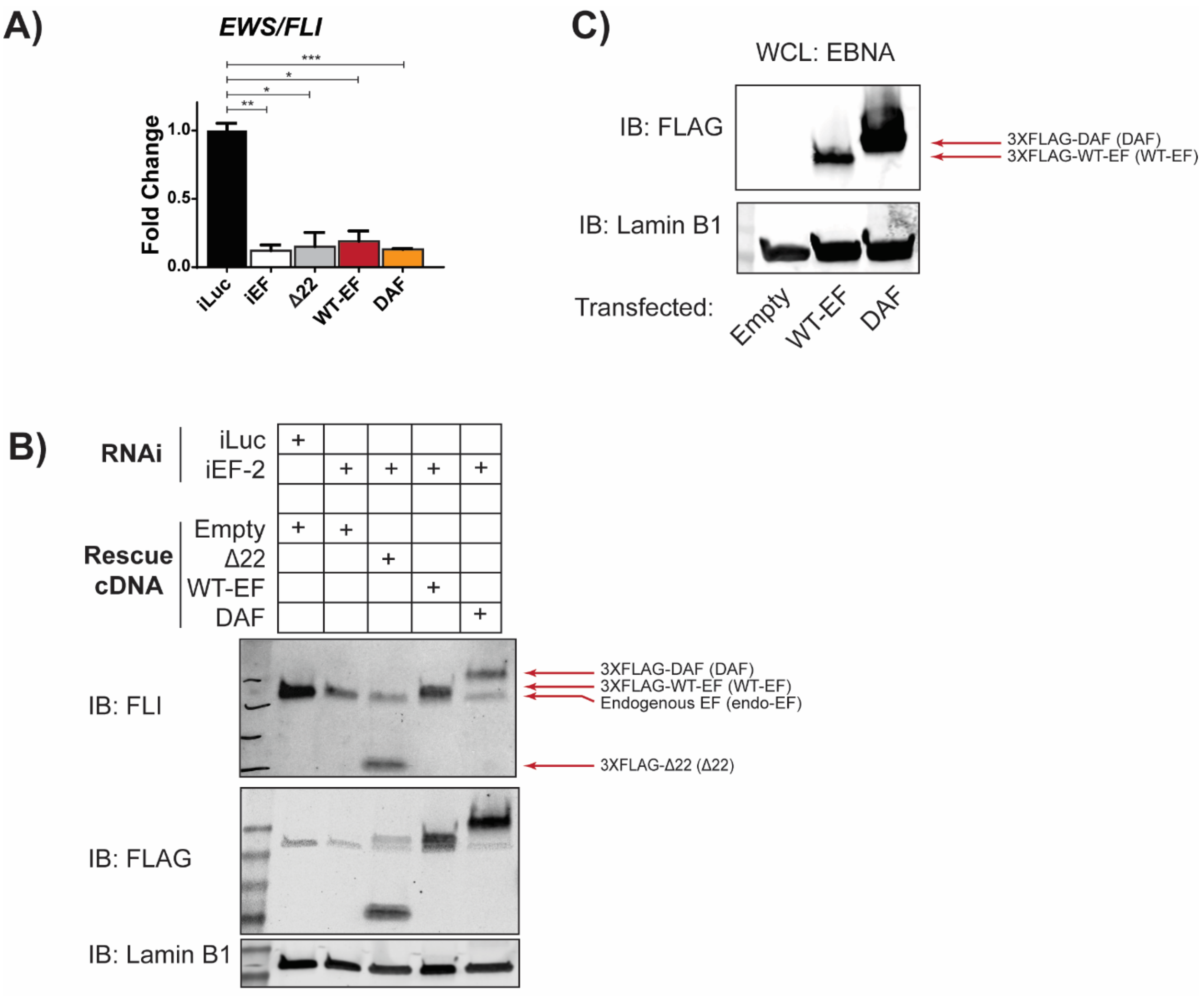

Supplementary Figure 2.

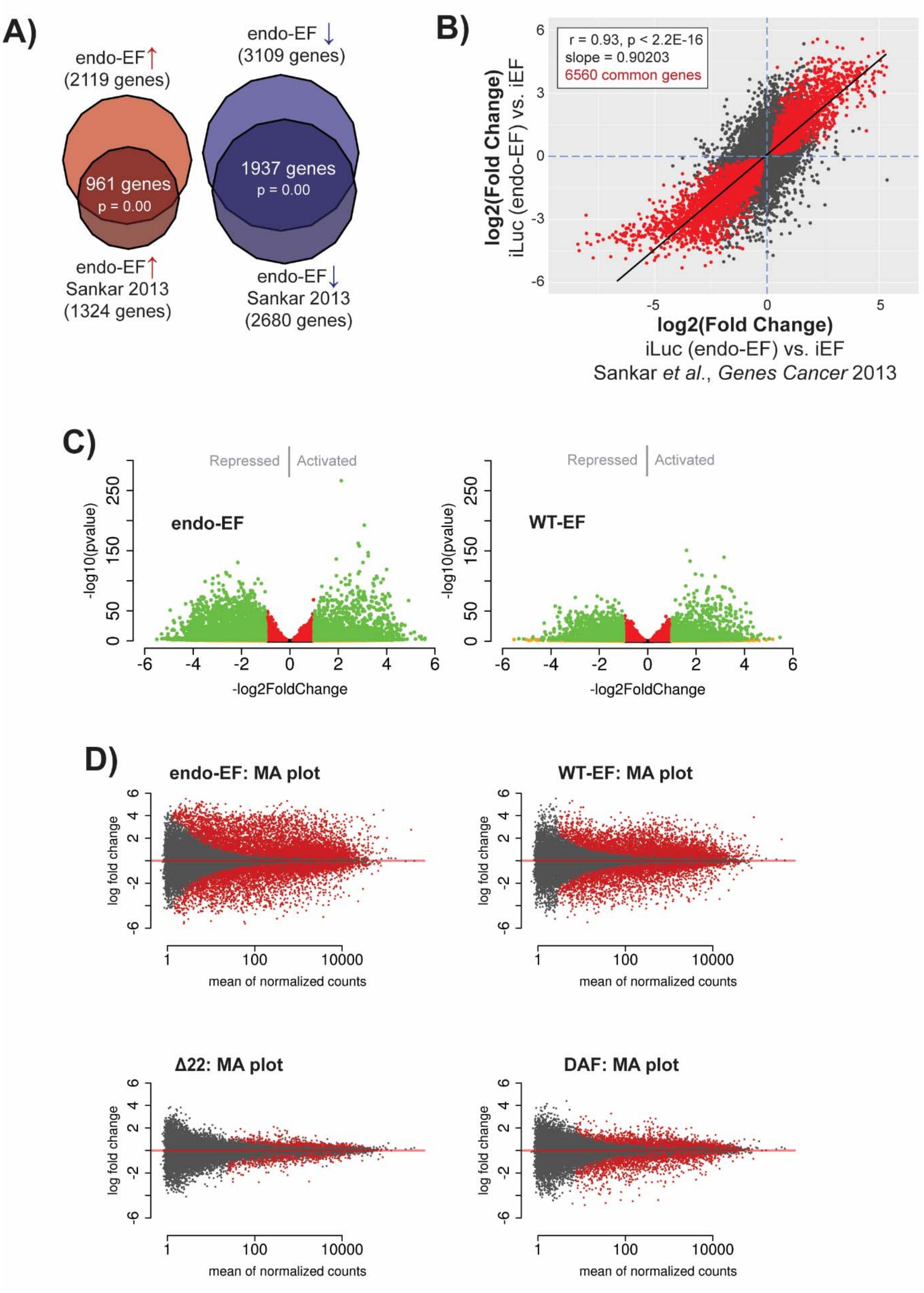

Supplementary Figure 3.

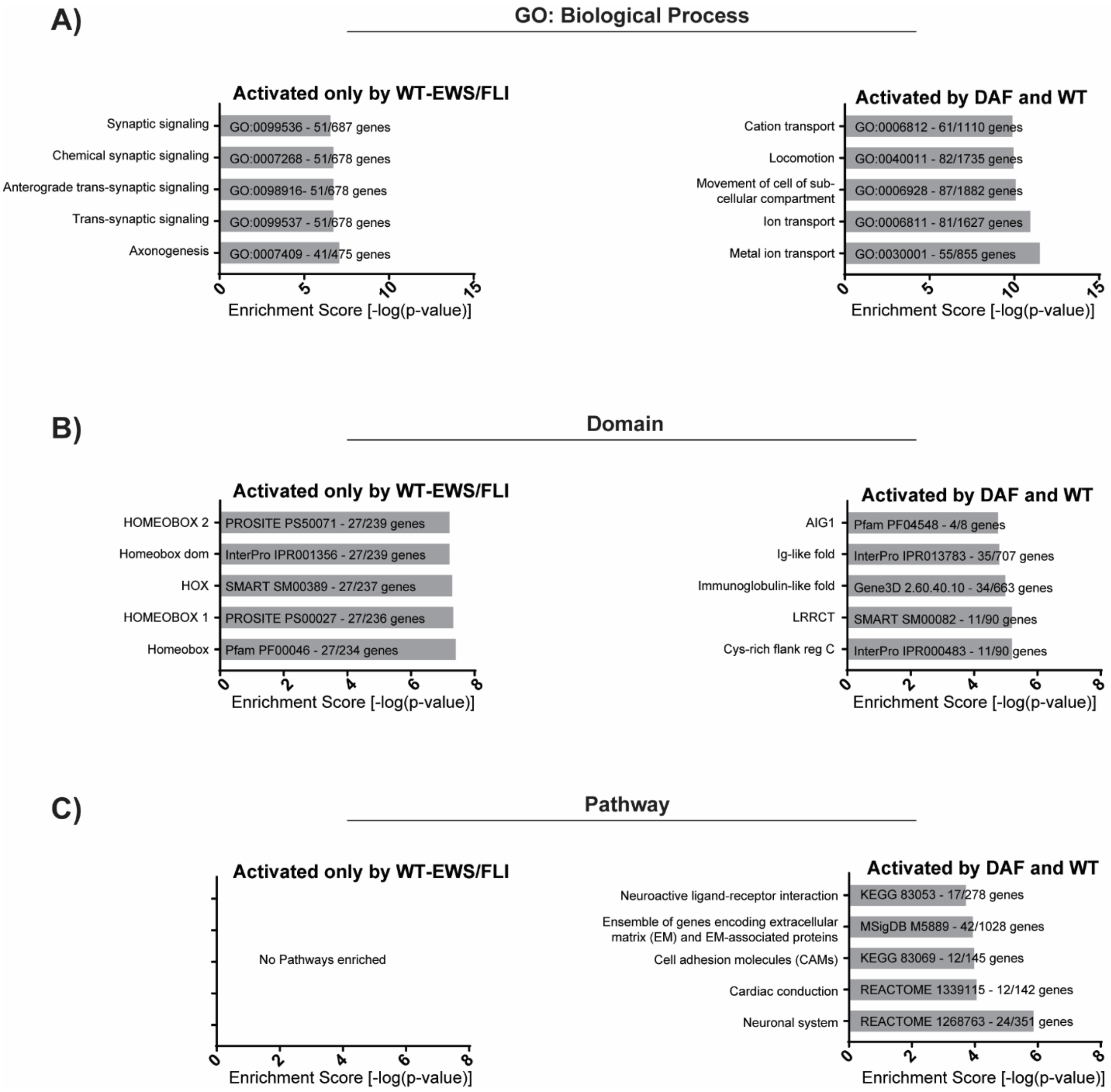

Supplementary Figure 4.

A) DAF-responsive and WT-EWS/FLI responsive

Rank	Motif	Best Match	%Targets	% BG	p-value	log P-pvalue
6	AATAAGGGTATA	SD0003.1_at_AC_acceptor/Jaspar	2.72%	0.14%	1e-11	-2.561e01
7	GAAGGTTAAT	ZNF652/HepG2-ChIP-Seq(Encode)	7.71%	1.90%	1e-10	-2.484e01
8	CGTGCCAGTGTG	PB0195.1_Zbtb3_2/Jaspar	3.40%	0.30%	1e-10	-2.445e01
9	GAGTGGGGAGAG	PB0107.1_Ascl2_2/Jaspar	9.75%	3.00%	1e-10	-2.411e01
10	CCGCGAGCAG	Ahr::Arnt/MA0006.1/Jaspar	10.88%	3.63%	1e-10	-2.330e01
11	TATACGCACT	PB0143.1_Klf7_2/Jaspar	9.07%	2.71%	1e-10	-2.296e01
12	TAATCGGGTT	Pax7/Myoblast-ChIP-Seq(GSE25064)	5.90%	1.21%	1e-9	-2.237e01
13	TATCTGATGA	MafB(bZIP)/HepG2-ChIP-Seq(GSE31477)	11.56%	4.23%	1e-9	-2.142e01
14	CTGCTAGGAGAA	STAT3/MA0144.4/Jaspar	6.12%	1.40%	1e-9	-2.062e01
15	GGGGGGTGCGGT	KLF16/MA0741.1/Jaspar	11.34%	4.32%	1e-8	-2.055e01

B) Only responsive to WT-EWS/FLI

Rank	Motif	Best Match	%Targets	% BG	p-value	log P-pvalue
6	CAAGGTTTATTT	Foxd3/MA0041.1/Jaspar	4.59%	1.01%	1e-11	-2.569e01
7	AGAAGCTCTGAC	POL008.1_DCE_S_I/Jaspar	3.16%	0.45%	1e-11	-2.564e01
8	GGGGTTCCCA	RBPJ/MA1116.1/Jaspar	22.09%	12.73%	1e-11	-2.546e01
9	GACCACTCTGGC	Bapx1/VertebralCol-ChIP-Seq(GSE36672)	1.87%	0.11%	1e-11	-2.539e01
10	GCAGCAGCCCAG	ZNF416/HEK293-ChIP-Seq(GSE58341)	19.08%	10.44%	1e-10	-2.516e01
11	ACGAGCGCTC	PB0099.1_Zfp691_1/Jaspar	18.36%	9.91%	1e-10	-2.505e01
12	GACTGGCGCC	PB0113.1_E2F3_2/Jaspar	9.76%	3.94%	1e-10	-2.441e01
13	CGCTGCAA	PB0091.1_Zbtb3_3/Jaspar	48.21%	35.97%	1e-10	-2.406e01
14	TGCCCCCCGACG	CTCFL/MA1102.1/Jaspar	2.73%	0.35%	1e-10	-2.391e01
15	ATCAGGCTAC	SIX1/MA1118.1/Jaspar	11.19%	5.02%	1e-10	-2.306e01

Supplementary Figure 5.

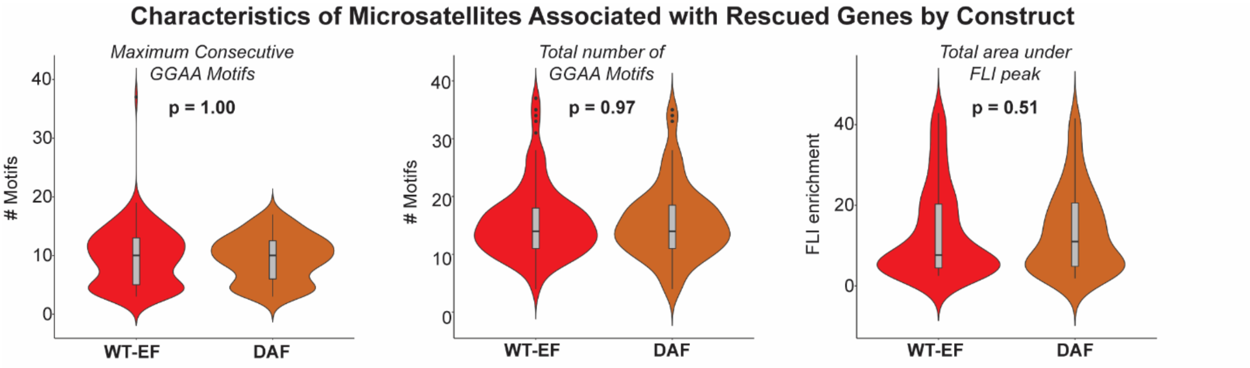

Supplementary Figure 6.

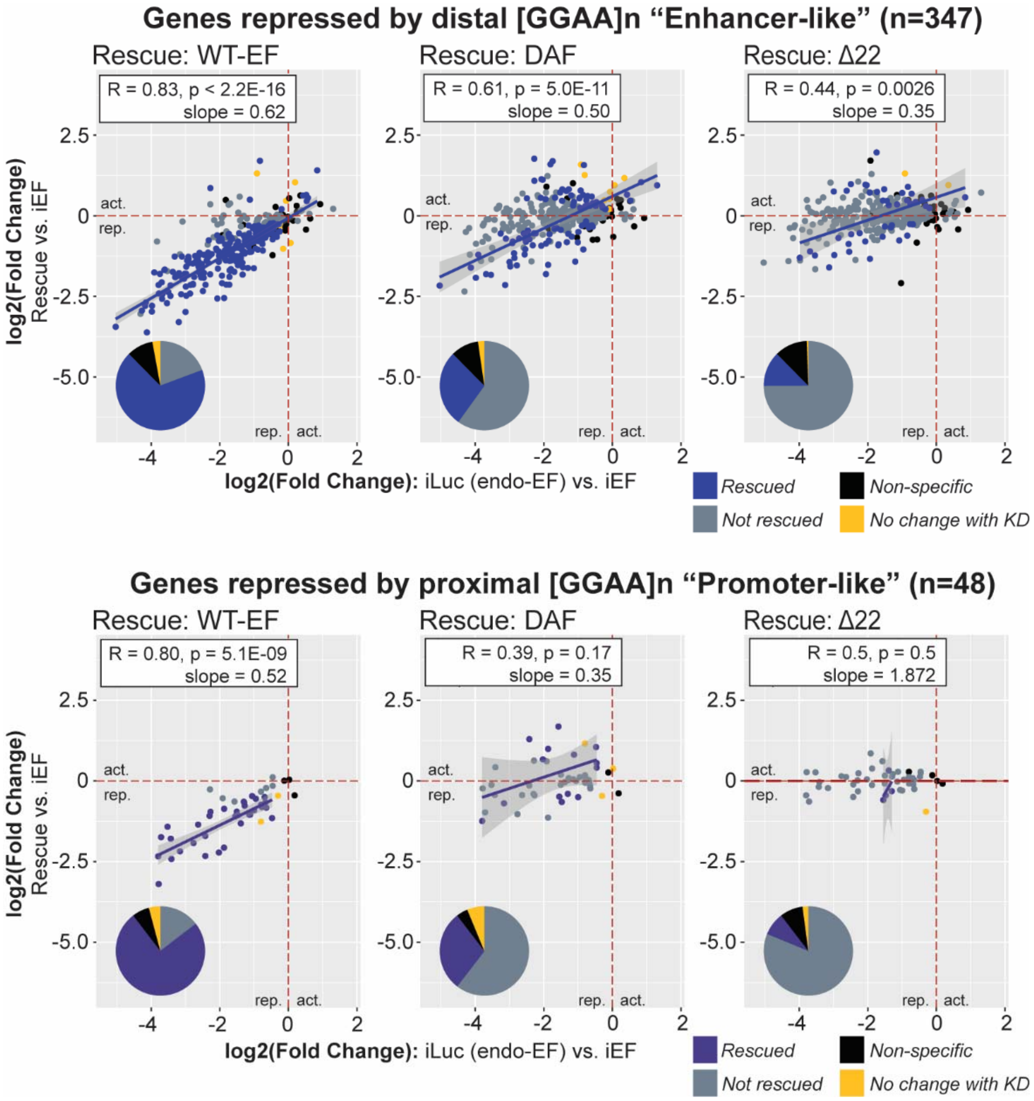

Supplementary Figure 7.

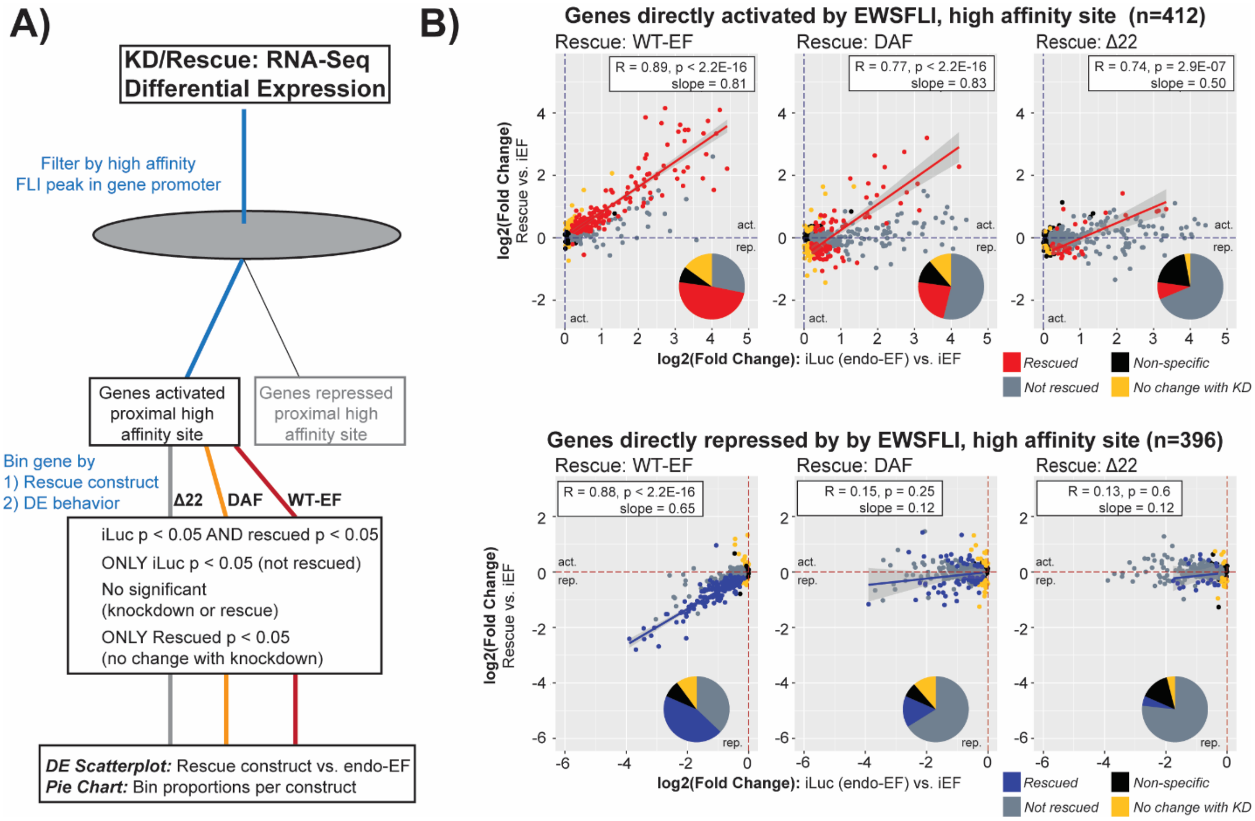

Supplementary Figure 8.

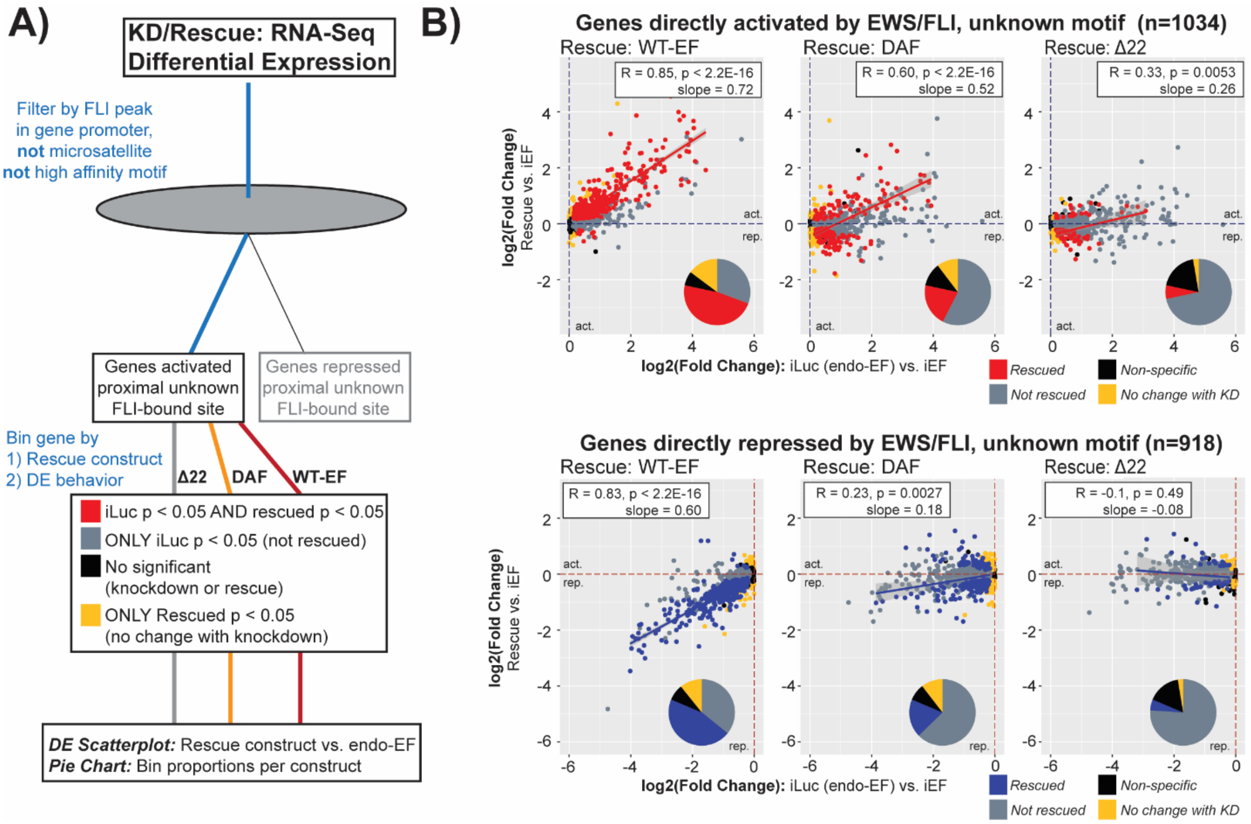

Supplementary Figure 9.

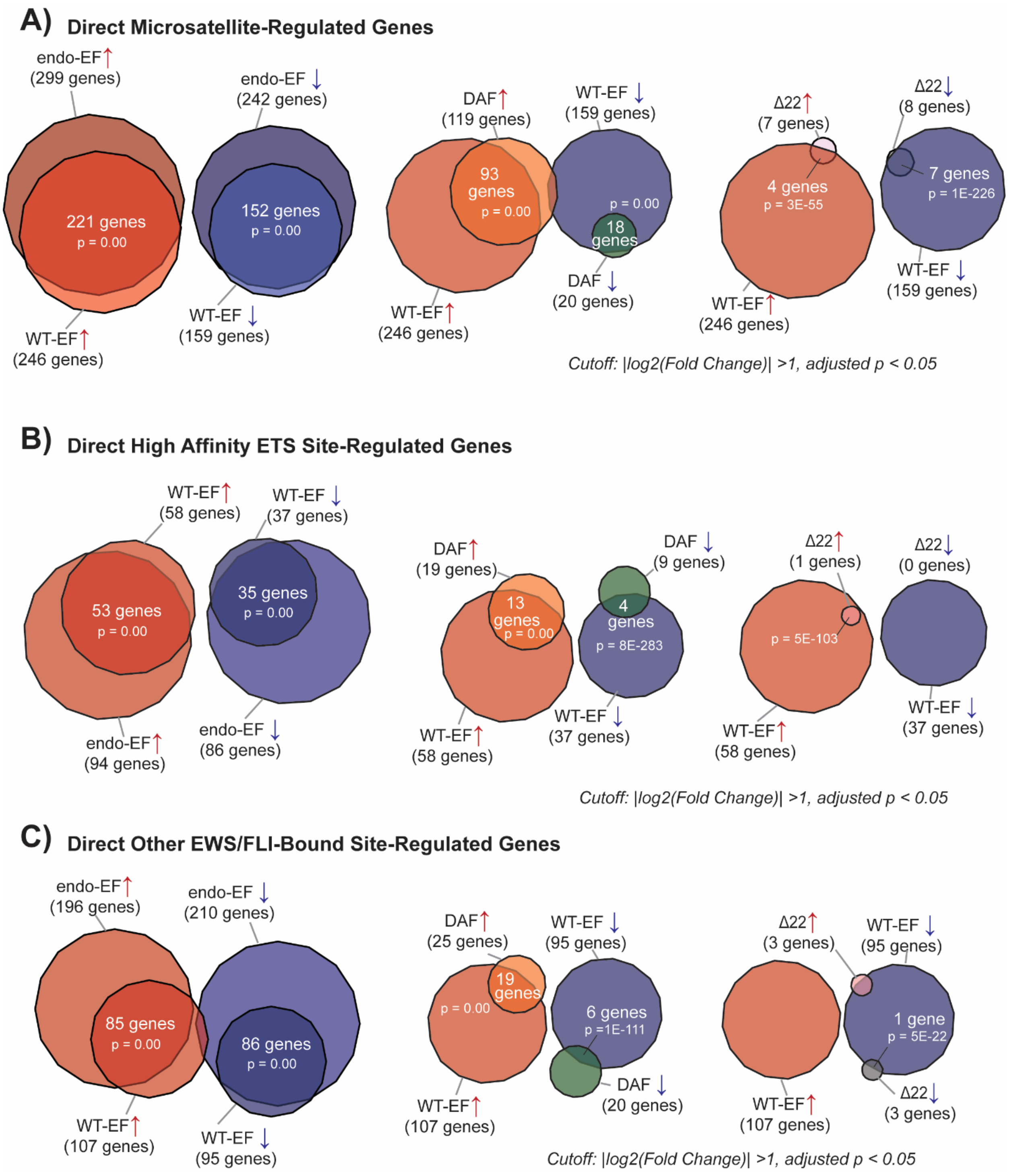

Supplementary Figure 10.

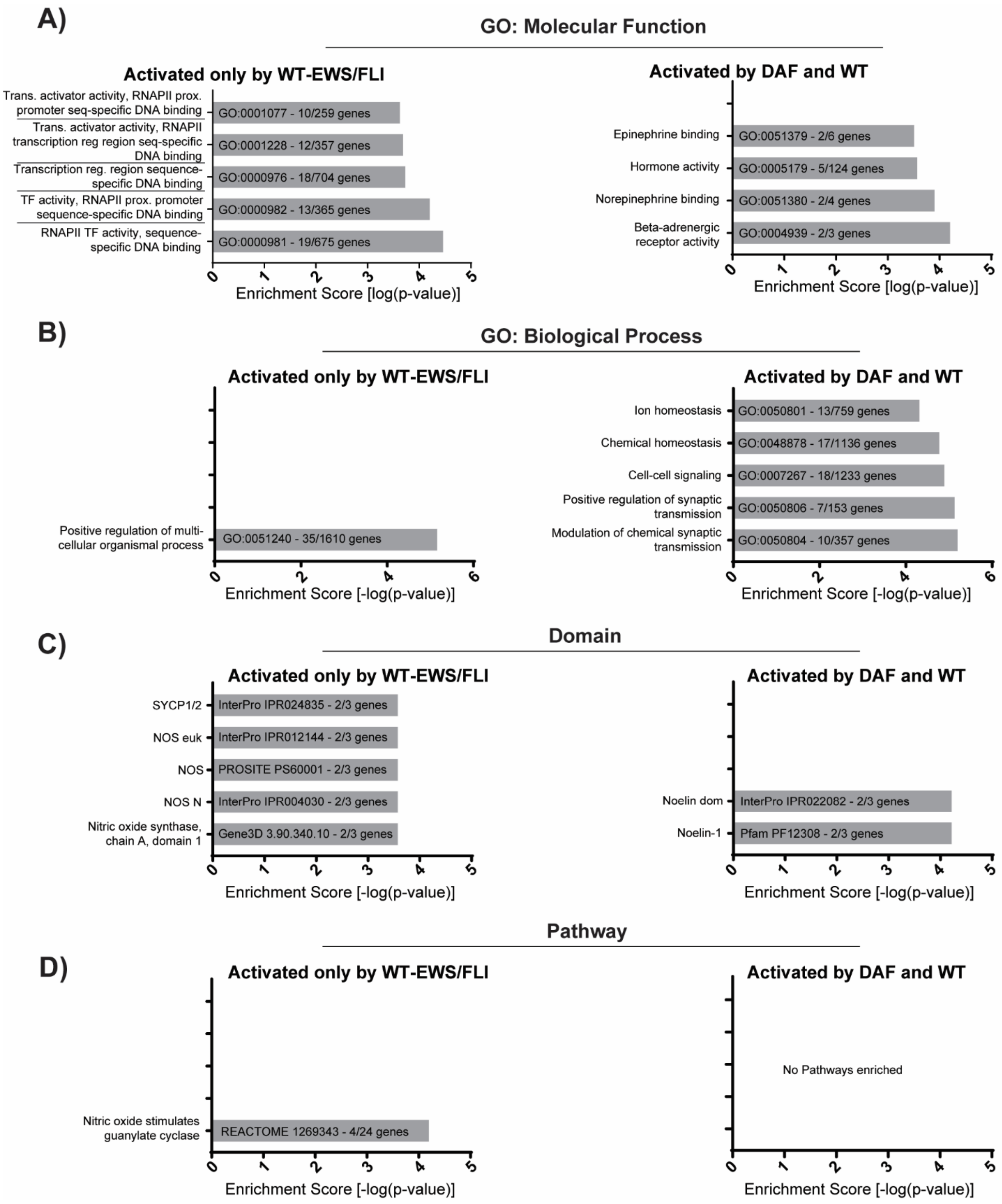

Supplementary Figure 11.

A) DAF-responsive and WT-EWS/FLI responsive - Direct Targets - HOMER Results

Rank	Motif	Best Match	%Targets	% BG	p-value	log P-pvalue
1	GTTAGCCGGA	PB0075.1_Sp100_1/Jaspar	16.84%	1.27%	1e-12	-2.978e01
2	AAATCTCTCTCC	Gfi1/MA0038.1/Jaspar	29.47%	5.98%	1e-11	-2.755e01
3	GAAGGTTAAT	Nkx6.1/Islet-ChIP-Seq(GSE40975)	21.05%	3.04%	1e-11	-2.537e01
4	AGCAATAAAACA	CDX2/MA0465.1/Jaspar	10.53%	0.42%	1e-10	-2.498e01
5	CTGTGAATTC	FOXH1/MA0479.1/Jaspar	42.11%	14.44%	1e-10	-2.350e01

B) Only responsive to WT-EWS/FLI - Direct Targets - HOMER Results

Rank	Motif	Best Match	%Targets	% BG	p-value	log P-pvalue
1	GCGAGCGCGGCT	SP4/MA0685.1/Jaspar	6.74%	0.45%	1e-10	-2.506e01
2	GGCTGGGTCCCA	ZNF692/HEK293-ChIP-seq(GSE58341)	8.81%	1.02%	1e-10	-2.414e01
3	AGATCCTGCAAG	SPDEF/VCaPChIP-seq(SRA014231)	5.18%	0.22%	1e-10	-2.403e01
4	AGAAATGGAG	Mef2c/GM12878-ChIP-Seq(GSE32465)	28.50%	11.14%	1e-10	-2.393e01
5	CGCTATTATG	Nkx2-5/MA0063.1/Jaspar	8.29%	0.89%	1e-10	-2.392e01

Supplementary Figure 12.

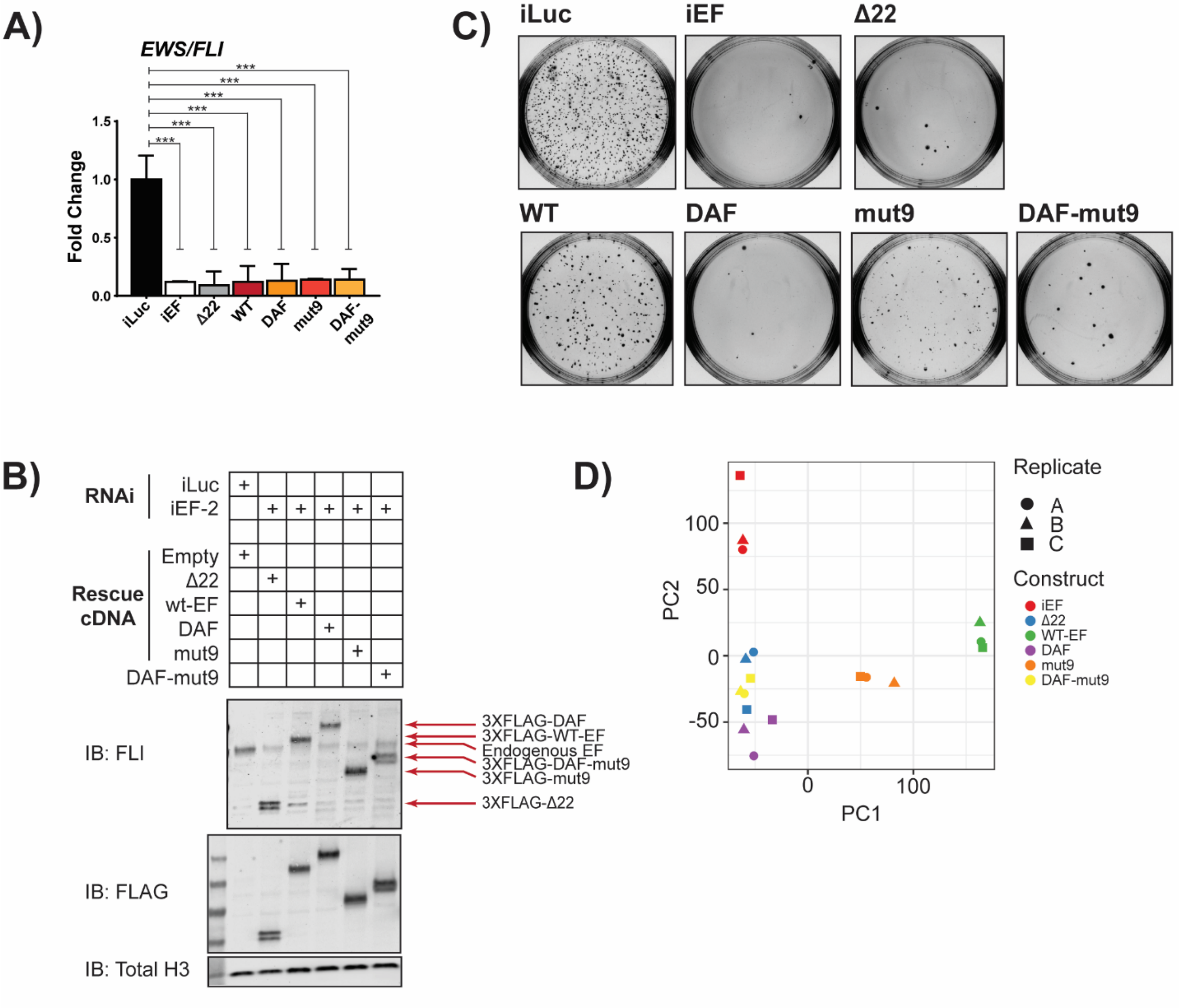

Supplementary Figure 13.

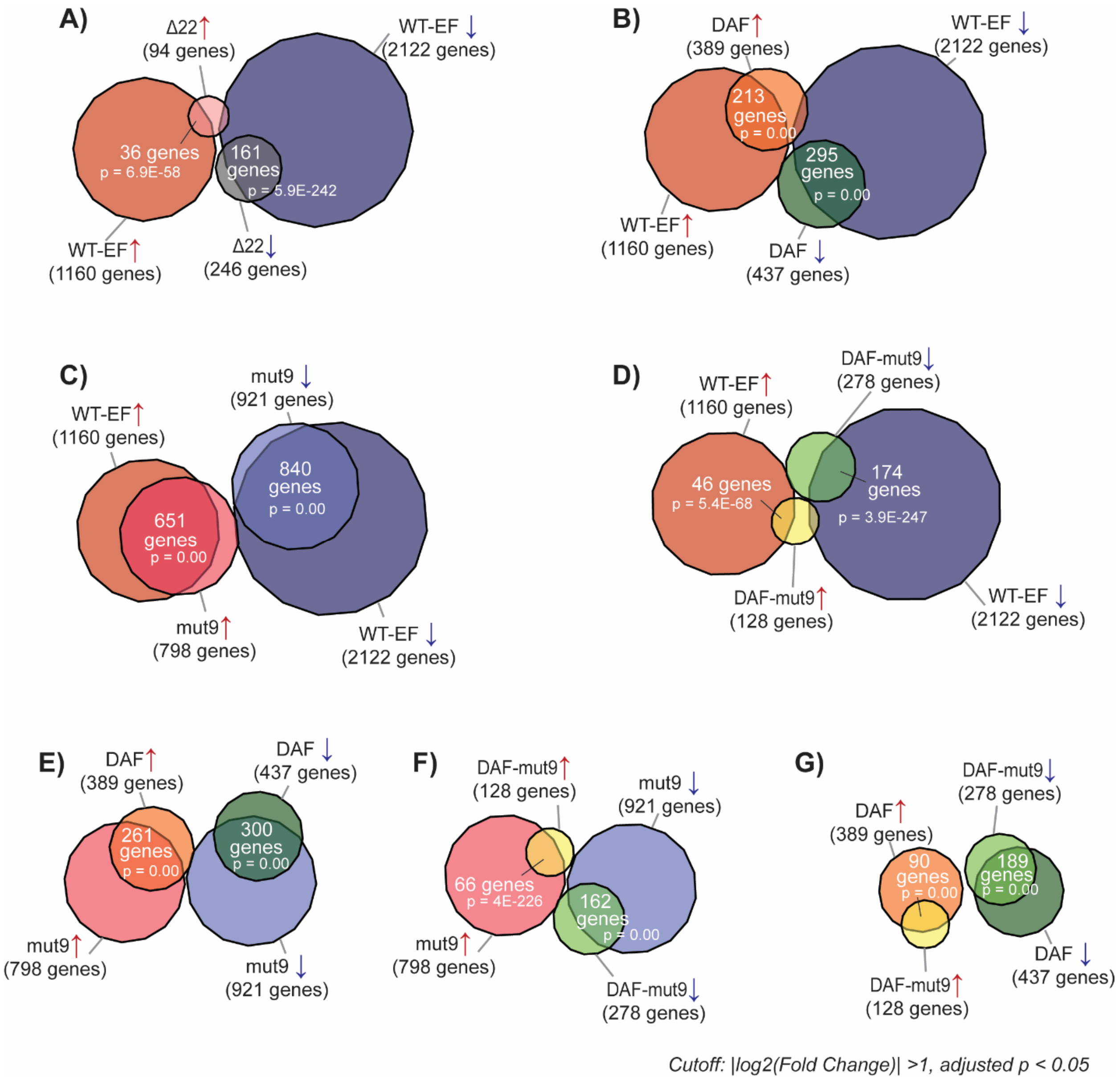

Supplementary Figure 14.

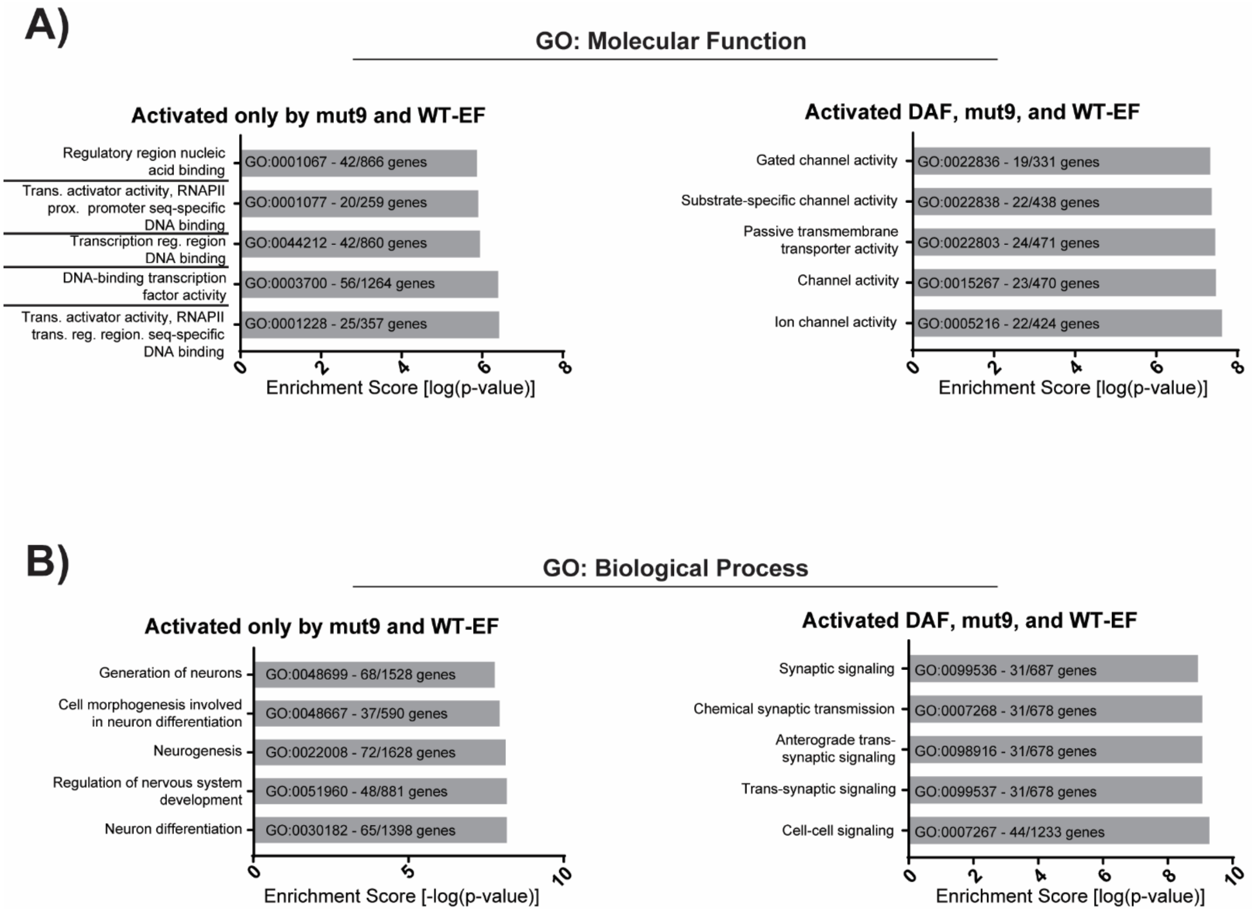

Supplementary Figure 15.

A) DAF-, mut9- and WT-EWS/FLI responsive

Rank	Motif	Best Match	%Targets	% BG	p-value	log P-pvalue
1	CGAGGGCCACTG	FXR(NR),IR1/Liver-ChIP-Seq(Chong et al)	6.85%	0.54%	1e-12	-2.971e01
2	GGGAGCCGCGCG	POL013.1_MED-1/Jaspar	15.32%	3.70%	1e-12	-2.916e01
3	CGTGAATCCCTC	PH0137.1_Pitx1/Jaspar	4.03%	0.13%	1e-11	-2.585e01
4	GAGATCTATT	HNF6(Homeobox)/Liver-ChIP-Seq(ERP000392)	9.27%	1.47%	1e-11	-2.582e01
5	GTTGCCTCCCCC	WT1(Zf)/Kidney-ChIP-Seq(GSE90016)	7.26%	0.82%	1e-11	-2.570e01
6	GGCGTAACTT	PB0143.1_Klf7_2/Jaspar	6.45%	0.64%	1e-10	-2.478e01
7	GACACATAAATT	HOXA5/MA0158.1/Jaspar	4.84%	0.29%	1e-10	-2.449e01
8	ATCAGTGTGTAG	ZSCAN4/MA1155.1/Jaspar	4.03%	0.16%	1e-10	-2.427e01
9	TGCTGTAAGCTG	PH0158.1_Rhox11_2/Jaspar	4.03%	0.18%	1e-10	-2.346e01
10	AAAAGACAATCA	Dux/MA0611.1/Jaspar	6.45%	0.72%	1e-10	-2.322e01

B) Only responsive to WT-EWS/FLI and mut9

Rank	Motif	Best Match	%Targets	% BG	p-value	log P-pvalue
1	GGTTGTGTGCGT	PB0208.1_Zscan4_2/Jaspar	5.42%	0.66%	1e-13	-3.160e01
2	AACTAGCACTCC	PH0168.1_Hnf1b/Jaspar	2.48%	0.07%	1e-12	-2.948e01
3	GTAGTAGTCA	Nr2e1/MA0676.1/Jaspar	7.45%	1.63%	1e-11	-2.717e01
4	TAGTTGTGGCTA	Klf4(Zf)/mES-ChIP-Seq(GSE11431)	2.26%	0.06%	1e-11	-2.668e01
5	ATAATGGATATA	NFAT(RHD)/Jurkat-ChIP-Seq(Jolma_et_al)	3.61%	0.33%	1e-11	-2.567e01
6	AAGAAAGAAAGG	PB0061.1_Sox11_1/Jaspar	9.93%	3.00%	1e-10	-2.524e01
7	CCAGGATAATAA	PH0151.1_Pou6f1_1/Jaspar	10.84%	3.60%	1e-10	-2.421e01
8	AATTATCGGG	PH0023.1_Dlx4/Jaspar	4.97%	0.84%	1e-10	-2.306e01
9	AACAACTATT	Arid5a/MA0602.1/Jaspar	21.90%	11.17%	1e-9	-2.301e01
10	CTCCCCACGC	MZF1/MA0056.1/Jaspar	32.28%	19.43%	1e-9	-2.275e01

Supplementary Figure 16.

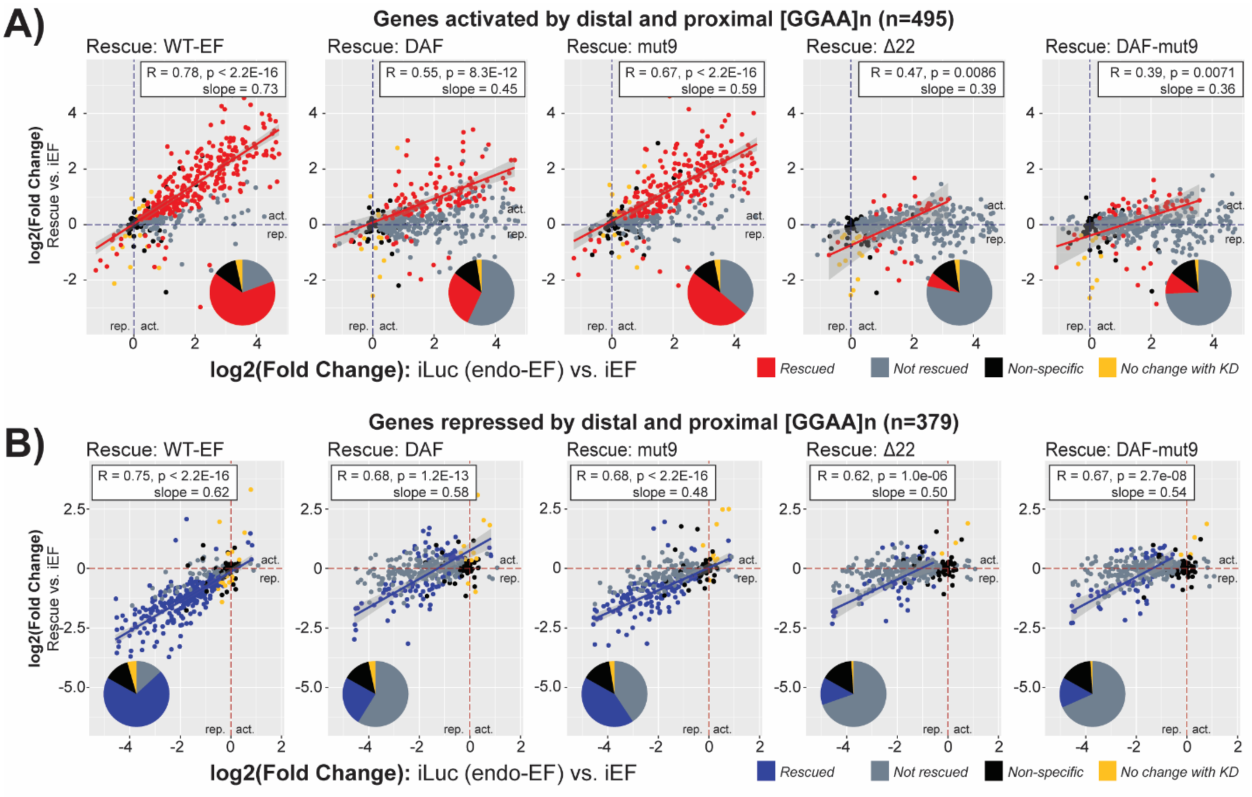

Supplementary Figure 17.

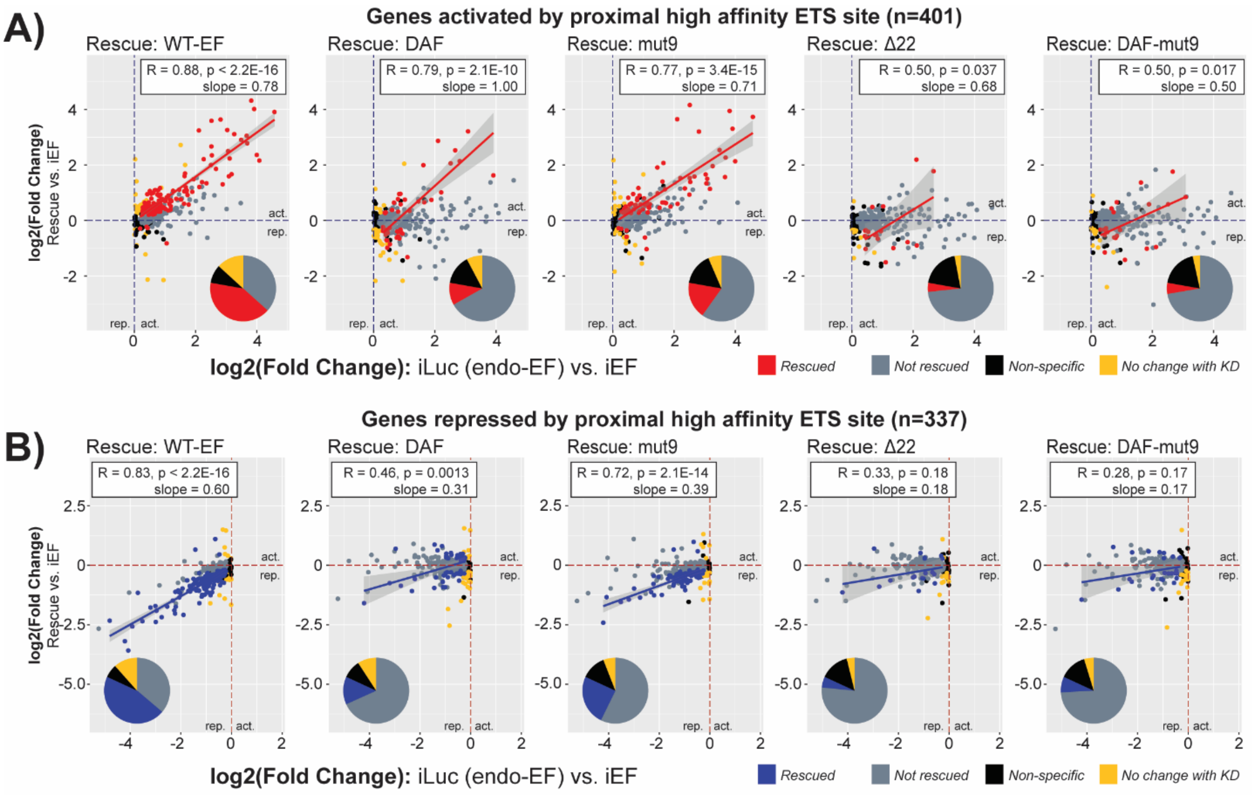

Supplementary Figure 18.

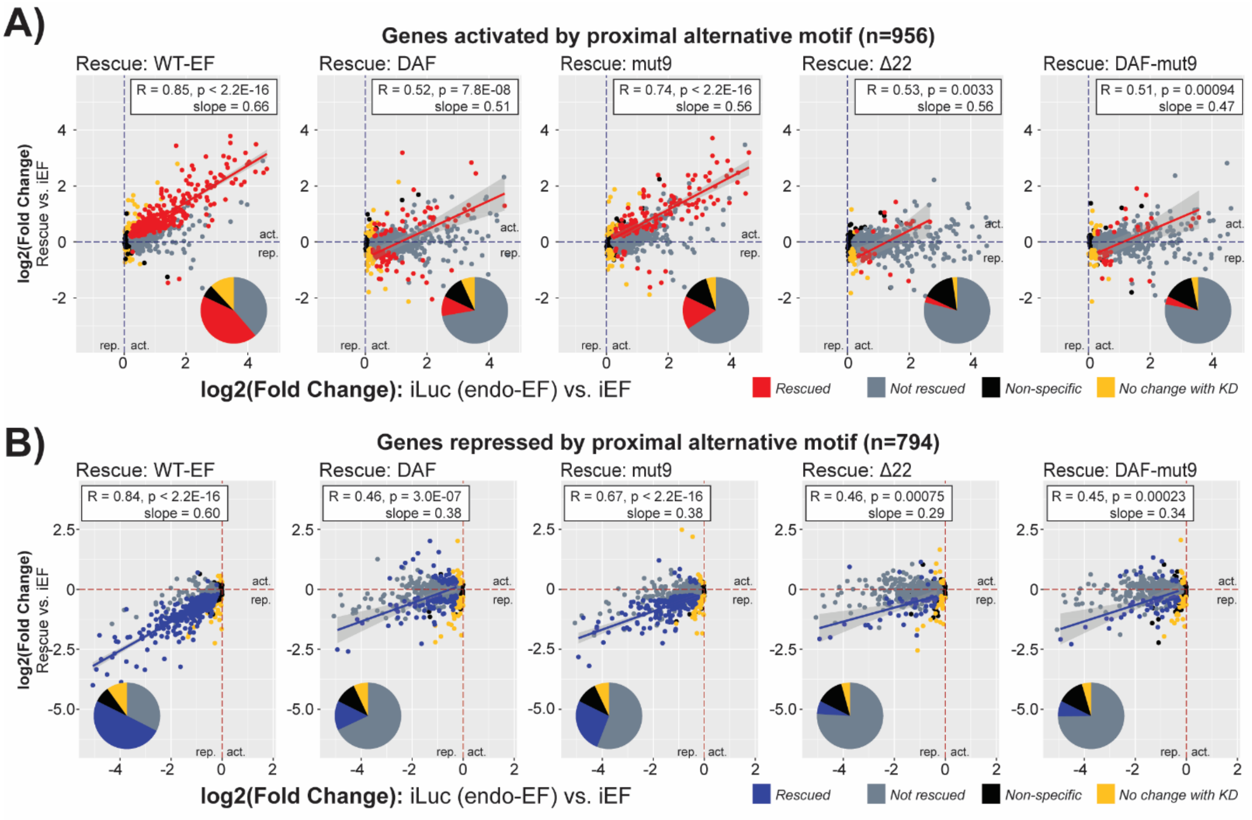

EWS/FLI **Raw Ct Data**

Tukey multiple comparisons of means "efkd2"

95% family-wise confidence level

Fit: aov(formula = EWSFLI ~ condition, data = efkd2)

$condition

	diff	lwr	upr	p adj
DAF-82/22	0.17333333	-0.32480485	0.67147152	0.8875018
DAF-82/22-82	0.02333333	-0.47480485	0.52147152	0.9999979
del22-82/22	0.72	0.22186181	1.21813819	0.0031886
iEF-82/22	0.24666667	-0.25147152	0.74480485	0.6321042
iLuc-82/22	-2.82333333	-3.32147152	-2.32519515	0
wtEF-82/22	0.22	-0.27813819	0.71813819	0.7363655
DAF-82/22-DA	-0.15	-0.64813819	0.34813819	0.9386378
del22-DAF	0.54666667	0.04852848	1.04480485	0.0274877
iEF-DAF	0.07333333	-0.42480485	0.57147152	0.998387
iLuc-DAF	-2.99666667	-3.49480485	-2.49852848	0
wtEF-DAF	0.04666667	-0.45147152	0.54480485	0.9998776
del22-DAF-82	0.69666667	0.19852848	1.19480485	0.0042468
iEF-DAF-82/2	0.22333333	-0.27480485	0.72147152	0.7237472
iLuc-DAF-82/2	-2.84666667	-3.34480485	-2.34852848	0
wtEF-DAF-82/	0.19666667	-0.30147152	0.69480485	0.818895
iEF-del22	-0.47333333	-0.97147152	0.02480485	0.0675206
iLuc-del22	-3.54333333	-4.04147152	-3.04519515	0
wtEF-del22	-0.5	-0.99813819	-0.00186182	0.0488764
iLuc-iEF	-3.07	-3.56813819	-2.57186182	0
wtEF-iEF	-0.02666667	-0.52480485	0.47147152	0.9999955
wtEF-iLuc	3.04333333	2.54519515	3.54147152	0

condition	EWSFLI
iLuc	2.456666667
iEF	5.546666667
del22	5.876666667
wtEF	5.453333333
DAF	5.376666667
82/22	5.296666667
DAF-82/22	5.186666667
iLuc	2.186666667
iEF	5.546666667
del22	6.216666667
wtEF	5.353333333
DAF	5.716666667
82/22	5.306666667
DAF-82/22	5.326666667
iLuc	2.776666667
iEF	5.536666667
del22	5.956666667
wtEF	5.743333333
DAF	5.316666667
82/22	5.286666667
DAF-82/22	5.446666667
